# Neutrophils promote tumor invasion *via* FAM3C-mediated epithelial-to-mesenchymal transition in gastric cancer

**DOI:** 10.7150/ijbs.79022

**Published:** 2023-02-21

**Authors:** Yaohui Wang, Xiang Li, Tiancheng Zhang, Fangyuan Li, Yuke Shen, Yani He, Qiang You, Yifen Zhang, Jing Zhai, Xuequan Yao, Lizong Shen

**Affiliations:** 1 Department of Surgical Oncology, Jiangsu Province Hospital of Chinese Medicine, Affiliated Hospital of Nanjing University of Chinese Medicine; Nanjing 210029, China.; 2 Department of Pathology, Jiangsu Province Hospital of Chinese Medicine, Affiliated Hospital of Nanjing University of Chinese Medicine; Nanjing 210029, China.; 3 Digestive Endoscopy Center, Jiangsu Province Hospital of Chinese Medicine, Affiliated Hospital of Nanjing University of Chinese Medicine; Nanjing 210029, China.; 4 Department of Geriatrics, the Second Affiliated Hospital, Nanjing Medical University; Nanjing 210003, China.; 5 Department of General Surgery, the First Affiliated Hospital, Nanjing Medical University; Nanjing 210029, China.

**Keywords:** Gastric cancer, Tumor-associated neutrophils, Lymph node metastasis, Epithelial-to-mesenchymal transformation, FAM3C.

## Abstract

In gastric cancer, lymph node metastasis (LNM) is the major metastasis route, and lymphatic invasion is the precursor of LNM. Tumor-associated neutrophils (TANs) promote LNM. However, the molecular mechanisms underlying TANs-mediated lymphatic invasion and/or LNM remain unclear. Herein, we revealed that high level of TANs was the independent risk factor for lymphatic invasion and LNM respectively, and lymphatic tumor cell-neutrophil clusters were positively correlated with LNM. Crosstalk between neutrophils and tumor cells was required for enhanced tumor cell invasiveness, endowing neutrophils to boost epithelial-to-mesenchymal transition (EMT) of tumor cells and in turn promoting LNM. Mechanically, tumor cells educated neutrophils via TGFβ1 to produce more FAM3C through Smad2/3 signaling activation, and FAM3C promoted tumor cell EMT through JNK-ZEB1/Snail signaling pathway. The crosstalk enhanced the affinity of neutrophils with tumor cells through interaction of integrins α6β1 and α6β4 with CD151. Furthermore, studies using tumor-bearing mice demonstrated that neutrophils were the important driver for gastric cancer tumorigenesis and invasiveness. The study clearly identifies the functional roles of TANs in promoting tumor invasion, and facilitates a better understanding of novel mechanisms responsible for LNM of gastric cancer, which provides potential targets for developing new strategies to prevent or treat LNM in gastric cancer.

## Introduction

Gastric carcinoma remains one of the most serious human health threats globally, being responsible for more than 700,000 deaths annually 1-3. Most patients have still been diagnosed at advanced stage, although early detection of gastric cancer keeps increasing. As the prominent metastasis route, lymph node metastasis (LNM) serves as the most prominent hallmark of disease progression and poor prognosis in gastric cancer patients 4-6. Elucidating the mechanisms underlying LNM should improve prevention and treatment for gastric cancer. Tumor LNM is a whole train of concurrent, usually overlapping processes 7. However, the current understanding of tumor LNM remains limited 8.

Gastric carcinoma is an extremely heterogeneous and aggressive cancer type. Tumor microenvironment (TME) comprises diverse cell components in addition to tumor cells, and tumor mesenchyma plays critical roles in carcinogenesis, tumor progression, recurrence, and drug resistance in gastric cancer and other cancer types 9-11. Unlike the conventional belief that neutrophils are just a bystander in tumor tissues, neutrophils in the TME, known as tumor-associated neutrophils (TANs), have been demonstrated to exert multifaceted effects on tumor development, including anti-tumoral roles initially, or transformation into cancer-promoting roles in which TANs synthesize or release multiple cytokines and display pro-tumoral manifestations 12-15. TANs also predict poor prognosis in several types of cancer 16. In gastric carcinoma, TANs have been shown to be associated with inflammation in tumor-draining lymph nodes (TDLNs) and systemic responses 17, and to portend clinical response to postoperative chemotherapy 18, 19. We have previously revealed that TANs promote LNM in early gastric cancer (EGC), and that TANs serve as the independent risk factor for LNM in addition to histological classification and lymphatic invasion in EGC 20. However, the mechanisms underlying TAN-mediated tumor invasion remain unclear.

Epithelial-to-mesenchymal transition (EMT) has been shown to be associated with various biological functions in cancer, such as tumorigenesis, progression, metastasis, and resistance to therapy 21-24. The Asian Cancer Research Group (ACRG) reported four major molecular subtypes for gastric carcinoma, including microsatellite instability (MSI), microsatellite stability (MSS)/EMT, MSS/TP53+ or MSS/TP53-. Among them, MSS/EMT subtype shows the worst prognosis 25. TANs-derived IL-17a promotes EMT of gastric cancer cells, and blockade of IL-17a signaling inhibits TANs-induced phenotypes in gastric cancer cells 26. However, whether EMT is involved in TANs-mediated tumor invasion remains unclear.

In this study, we performed comprehensive clinicopathologic analyses of 257 patients with gastric adenocarcinoma confined in submucosa (T1b tumor) to ascertain the roles of TANs in tumor invasion. Furthermore, we investigated the molecular mechanisms underlying enhanced-tumor invasiveness mediated by the crosstalk between neutrophils and gastric cancer cells. Our studies identify functional roles of TANs in promoting tumor invasion, and facilitate a better understanding of mechanisms responsible for LNM of gastric carcinoma.

## Materials and Methods

### Patients

A successive series of 257 patients with T1b gastric adenocarcinoma from January 2011 to December 2020 in the Affiliated Hospital of Nanjing University of Chinese Medicine, who underwent radical gastrectomy with curative intent, were enrolled. All patients had not received preoperative therapy. The clinicopathologic data were collected. All the pathological characteristics were reviewed by two professional pathologists. The samples were obtained following informed consent in line with an established protocol approved by Institutional Review Board of Nanjing University of Chinese Medicine. This study was also in agreement with the Declaration of Helsinki.

### Tumor-associated neutrophil analysis

Tumor-associated neutrophils (TANs) were determined by hematoxylin & eosin (H & E) staining or immunohistochemistry (IHC) with anti-CD66b (Abcam, Cambridge, UK). Lymphatic invasion was determined by H & E staining, and IHC with anti-D2-40 (Dako, Carpinteria, USA) was used to differentiate lymphatic invasion and vascular invasion. Neutrophils in cancer embolus were determined with H & E staining.

### Immunohistochemistry assay

Immunohistochemistry (IHC) assay was conducted *as per* the standard protocols. The monoclonal antibodies used for the analyses were as following: rabbit anti-CD66b (Abcam, Cambridge, UK), mouse anti-D2-40 (Dako, Carpinteria, USA), mouse anti-E-cadherin (E-cad) (Dako, Carpinteria, USA), rabbit anti-family with sequence similarity 3 member C (FAM3C) (Abcam, Cambridge, UK), rabbit anti-integrin α6 (Abcam, Cambridge, UK) and rabbit anti-CD151 (Affinity Biosciences, Cincinnati, USA) (for human tissue specimens), mouse anti-E-cad (Cell Signaling Technology, MA, USA), rabbit anti-vimentin (Vim) (Cell Signaling Technology, MA, USA) (for mouse allograft). Integrin α6 (ITGA6) and CD151 were detected using dual-color immunostaining in gastric tumor tissues. The expression intensity of E-cad or Vim was evaluated with mean density (MD) using Image-Pro Plus 6.0 image analysis software (Media Cybernetics, America), and MD means integral optical density (IOD) SUM divided by area SUM.

### Cell lines and cell culture

Human gastric adenocarcinoma cell lines, MKN28 (CBTCCCAS, Shanghai, China), MKN45 (CBTCCCAS, Shanghai, China), and mouse gastric cancer cell line, MFC (CBTCCCAS, Shanghai, China), were cultured in the complete RPMI1640 (GIBCO, VA, USA) supplemented with 10% fetal bovine serum (Hyclone, Logan, UT, USA).

### Isolation, culture and flow cytometry analyses of peripheral neutrophils

Peripheral neutrophils were isolated from 20 ml whole blood samples of healthy donors or patients with gastric cancer using human peripheral blood neutrophil separation reagent kit (Solarbio, Beijing, China) *as per* the recommended protocols. The cells were washed with red blood cell lysis buffer (BD Biosciences, NJ, USA), centrifuged and washed with PBS. The isolated neutrophils were cultured in complete RPMI1640 (GIBCO, VA, USA). To determine the purity and viability, the isolated neutrophils were washed and resuspended in stain buffer (BD Biosciences, NJ, USA) for flow cytometry (FCM) analysis (BD FACSAria II flow cytometer, BD Biosciences, Franklin Lakes, NJ, USA). FITC anti-human CD45 antibody, APC anti-human CD66b antibody, and Fixable Viability Stain 780 (BD Biosciences, Franklin Lakes, NJ, USA) were used respectively.

### Cell migration and invasion assay

Wound-healing assay was applied to assay cell migration. Tumor cells were seeded and cultured in a 6-well plate. When a confluent monolayer was formed, a sterile plastic tip was used to scratch on the monolayer of cells. Pictures were taken with a microscope at the specified time points to monitor the migration distance. Migration was quantified as a percentage of wound closure. Transwell assay was performed to ascertain cell invasion using Transwell apparatus (Corning Life Sciences, Corning, NY, USA) with diluted matrigel (BD Bioscience, CA). MKN28 or MKN45 cells (2 × 10^5^) in 200 μl serum-free medium were seeded into the upper chamber. Conditioned mediums (CMs) of different groups were added to the upper chamber. After incubation at 37 °C for 24 h, the cells were fixed with 4% paraformaldehyde, stained by crystal violet, and then photographed under a microscope.

### RNA sequencing analysis of neutrophils

Total RNA was isolated and reversely transcribed into cDNA to generate an indexed Illumina library, followed by sequencing at the Beijing Genomics Institute (Beijing, China) using a BGISEQ-500 platform. Significant differential expression of a gene was defined as a > 2-fold expression difference *vs* the control with an adjusted *P* value less than 0.05. A heat map was analyzed by Gene Ontology (GO) using Cluster software and visualized with Java Treeview. Differentially expressed genes (DEGs) were analyzed by GO using the AMIGO and DAVID software. The enrichment degrees of DEGs were analyzed using Kyoto Encyclopedia of Genes and Genomes annotations (KEGG).

### Western blotting assay

Expression of the indicated molecules was determined using the western blotting assay. The antibodies were as following: rabbit polyclonal anti-FAM3C antibody (Abcam, Cambridge, UK), rabbit polyclonal anti-CD151 antibody (Affinity Biosciences, Cincinnati, USA), rabbit monoclonal anti-E-cad antibody, rabbit monoclonal anti-ZO-1 antibody, rabbit monoclonal anti-Claudin-1 antibody, rabbit monoclonal anti-Vim antibody, rabbit monoclonal anti-N-cadherin antibody, rabbit monoclonal Snail antibody, rabbit monoclonal ZEB1 antibody, rabbit monoclonal TWIST1 antibody, rabbit monoclonal anti-phosphorylated AKT (anti-p-AKT) antibody, rabbit monoclonal anti-p-Erk1/2 antibody, rabbit monoclonal anti-p-JNK antibody (Cell Signaling Technology, MA, USA). Relative levels were quantified and normalized with GAPDH in the same sample with density analysis.

### Immunofluorescence assay

Tumor cells on coverslips were fixed with 4% paraformaldehyde for 15min. Then, in PBS containing 10% bovine serum albumin, the sections were blocked at room temperature for 2 h. After blocking, samples were incubated with primary antibodies specific for mouse anti-E-cad (Abcam, Cambridge, UK), mouse anti-Vim (Abcam, Cambridge, UK) overnight at 4℃. Fluorescent secondary antibody was carried out for 1 h at room temperature. Cell nuclei were counterstained with DAPI (Sigma-Aldrich, MO, USA). Images were acquired on a Zeiss LSM510 confocal microscope (Oberkochen, Germany).

### Enzyme-linked immunosorbent assay

Human FAM3C enzyme-linked immunosorbent assay (ELISA) Kit (Fine Test, Wuhan, China) was used to measure FAM3C levels in the CMs of the isolated neutrophils and the tumor-educated neutrophils. Assaying procedures were performed according to the recommended protocols. Each experiment was repeated at least three times.

### Real-time quantitative PCR

The mRNA level of each gene was analyzed using real-time quantitative PCR (qPCR). The primer sequences were integrin α6-F CGAAACCAAGGTTCTGAGCCCA, integrin α6-R CTTGGATCTCCACTGAGGCAGT; integrin β1-F CCTACTTCTGCACGATGTGATG, integrin β1-R CCTTTGCTACGGTTGGTTACATT; integrin β4-F GCAGCTTCCAAATCACAGAGG, integrin β4-R CCAGATCATCGGACATGGAGTT.

### Co-immunoprecipitation

CD151-Flag, integrin α6-His, integrin β1-HA, integrin β4-HA expression plasmids (Sino Biological Inc., Beijing, China) were transfected into HEK-293 cells simultaneously. Cells were collected and lysed in co-immunoprecipitation (co-IP) buffer (20 mM Tris, pH 7.5, 150 mM NaCl, 1% Triton X-100, and 1 mM EDTA) with protease inhibitors (Roche Applied Science, Mannheim, Germany) for 30 min on ice. Then the supernatant was taken following 10 min centrifugation (12, 000×g, 4 °C), and the supernatant was incubated with primary anti-Flag, anti-His, anti-HA (Cell Signaling Technology, MA, USA), following rocking gently overnight at 4 °C, and antibody-protein precipitates were pulled down by Dynabeads Protein G (Invitrogen, CA, USA). Then beads were washed extensively and proteins were eluted before subjected to analysis by western blotting.

### Establishment of subcutaneous or abdominal allograft tumor model of gastric cancer in C57BL/6 Mice

Animal studies were approved by the Animal Management and Use Committee of Nanjing University of Chinese Medicine. Four-week-old male C57BL/6 mice (Vitalriver, Nanjing, China) were randomly divided into two groups, subcutaneous allograft group and abdominal allograft group, and each contained 12 mice. After digestion and washing, MFC cells were inoculated into the hypochondrium subcutaneously or into abdominal cavity (1.0 × 10^6^ cells *per* mouse). Each group was further randomly divided into two subgroups, neutrophil deletion group and control group, each containing 6 mice. For neutrophil depletion, mouse anti-Ly6G antibody (Bio X cell, West Lebanon, USA) was daily administrated intraperitoneally after tumor cell inoculation (25 μg *per* day), while mice in the control group received equivalent dose of IgG2a isotype. Two weeks later, these mice were euthanized, and the subcutaneous or abdominal tumors were collected. Subcutaneous tumor volume was calculated by width × length × (width +length) / 2.

### Counting of neutrophils in peripheral blood in mice

Counting of neutrophils in peripheral blood in mice was performed with FCM. Peripheral whole blood specimens of mice (30 μl per mouse) were directly stained with antibodies of anti-CD45, anti-CD11b and anti-Ly6G (Abcam, Cambridge, UK) according to standard protocols. Neutrophils were identified as CD45^+^CD11b^+^Ly6G^+^ cells.

### Statistical analysis

Differences and relationships between groups with continuous or categorical variables were statistically compared with Student t, χ^2^, Fisher's exact test and Spearman correlation analysis using SPSS software (version 22.0; SPSS Inc., Chicago, IL). A multivariate logistic regression analysis was performed to identify independent risk factors. All experiments were repeated at least three times. All values in the text and graph deviate from the mean standard. *P* value less than 0.05 was considered significant.

## Results

### TANs promote tumor invasion in gastric cancer

**Figure 1:**
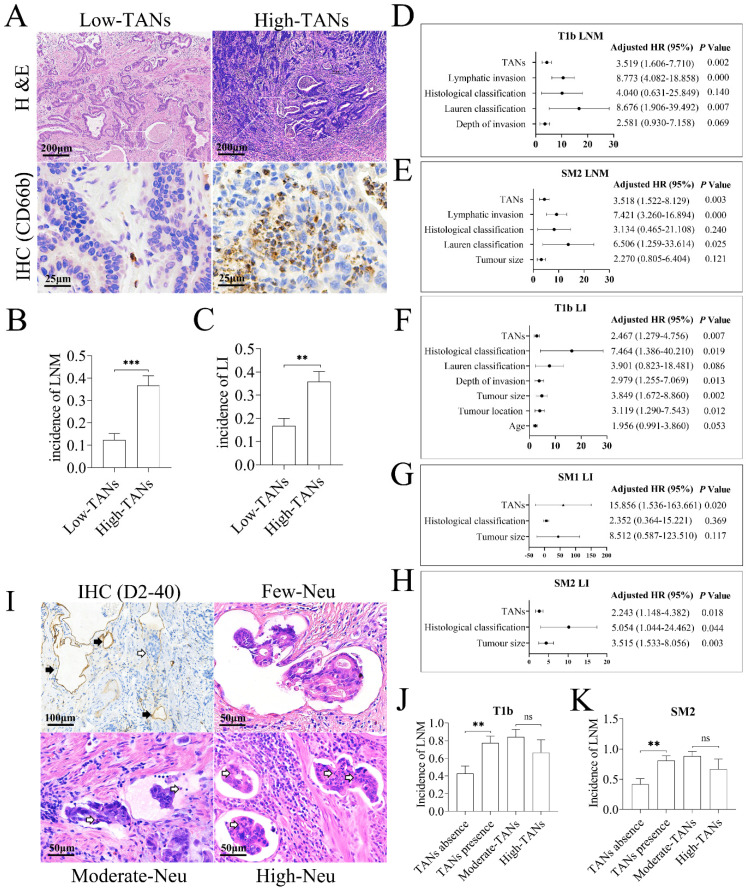
** TANs promote tumor invasion in gastric cancer.** (**A**) TANs were detected in the tumor stroma of nearly all T1b gastric cancer tissues by H & E staining and IHC. In low-TANs group, TANs were rare and occasionally present, and the enlarged frame showed few neutrophils with CD66b staining. In high-TANs group, more than 10 neutrophils *per* HPF were detected, and the enlarged frame showed multiple neutrophils with CD66b staining. (**B**) The incidence of LNM in high-TANs group was much higher than that in low-TANs group (*P* = 0.000). (**C**) The incidence of lymphatic invasion (LI) in high-TANs group was much higher than that in low-TANs group (*P* = 0.001). (**D**) The multivariate analysis indicated that Lauren classification, lymphatic invasion and high TANs are the independent risk factors for LNM in T1b tumors. (**E**) Lauren classification, lymphatic invasion and high TANs were the independent factors for LNM in SM2 tumors. (**F**, **G**, **H**) TANs served as one of the independent risk factors for lymphatic invasion (LI) in T1b, SM1 and SM2 tumors respectively. (**I**) Neutrophils could be detected in about half of lymphatic cancer emboli in T1b tumors. IHC with anti-D2-40 showed the lymphatics (black arrow) in tumor tissues, and blank arrows show the cancer embolus and the neutrophils in cancer emboli. (**J**, **K**) The incidence of LNM in patients with neutrophils in lymphatic cancer emboli was significantly increased with regard to that in patients without neutrophils in cancer emboli in either T1b tumors (**J**) or in SM2 tumors (**K**). However, the neutrophil numbers in emboli had no influence on LNM (**J**, **K**). (**, *P* < 0.01; ***, *P* < 0.001) (Neu, neutrophil)

Our previous study has demonstrated that TANs promote LNM in EGC 20. However, LNM of EGC mainly occurs in submucosal (T1b) tumors, and lymphatic invasion, identified by detection of cancer embolus in lymphatic vessels and recognized as a hallmark of poor prognosis in gastric cancer 27, 28, is the precursor for LNM. To investigate the functional role of TANs in tumor invasion, the clinicopathologic data of the enrolled 257 patients with T1b tumor were reviewed retrospectively. As shown in **Figure 1A**, neutrophils could be detected in almost all T1b cancer tissues, and mainly located in mucosa. Based on the median TAN number of 10 *per* non-overlapping high-power field in primary tumor tissues, these patients were divided into high- and low-TANs groups 29, and 46.7% of cases constituted the high-TANs group (**Table 1**). High TAN levels were associated with macroscopic type (*P* = 0.026), Lauren classification (*P* = 0.022) and histological classification (*P* = 0.004). Importantly, the incidence of LNM (*P* = 0.000) and lymphatic invasion (*P* = 0.001) in high-TANs group was much higher than that in low-TANs group respectively (**Figure 1B**, **1C**). The risk factors for LNM in T1b tumors were also analyzed. The average number of removed regional lymph nodes was 24.8 *per* case (range from 12 to 74). The incidence of LNM was 23.7% in the whole patients, and it was much higher in SM2 tumors (tumor infiltration into the deep submucosal layer, ≥ 500 μm from the muscularis mucosae) (27.7%) than that in SM1 tumors (tumor infiltration confined in the superficial submucosal layer, < 500 μm from the muscularis mucosae) (13.04%) (*P* = 0.014). Univariate analysis revealed that depth of invasion (*P* = 0.014), Lauren classification (*P* = 0.000), histological classification (*P* = 0.000), lymphatic invasion (*P* = 0.000), and high TANs (*P* = 0.000) were positively associated with LNM in these 257 patients (**Table s1A**). However, multivariate analysis indicated that Lauren classification (*P* = 0.007), lymphatic invasion (*P* = 0.000) and high TANs (*P* = 0.002) were independent risk factors (**Figure 1D**, **Table s1B**). Stratification analyses according to SM1 or SM2 tumors indicated that Lauren classification (*P* = 0.000), histological classification (*P* = 0.001), lymphatic invasion (*P* = 0.001) and high TANs (*P* = 0.009) were associated with LNM in SM1 diseases (**Table s1C**); however, no independent risk factor was observed (**Table s1D**). In SM2 tumors, the risk factors included tumor diameter (*P* = 0.049), Lauren classification (*P* = 0.000), histological classification (*P* = 0.017), lymphatic invasion (*P* = 0.000) and high TANs (*P* = 0.000) (**Table s1E**), whereas Lauren classification (*P* = 0.025), lymphatic invasion (*P* = 0.000) and high TANs (*P* = 0.003) were independent risk factors for LNM (**Figure 1E**, **Table s1F**).

Furthermore, the incidence of lymphatic invasion was 25.7% in T1b tumors, and it was much higher in SM2 tumors (30.3%) than that in SM1 diseases (13.04%) (*P* = 0.006). Notably, TANs served as an independent risk factor for lymphatic invasion in T1b, SM1 and SM2 tumors (**Figure 1F**, **1G** and **1H**, **Table s2A-s2C**). Given that lymphatic invasion or high TANs was the independent risk factor for LNM in EGC, especially in T1b disease, we next explored the relevance of neutrophils in lymphatic cancer embolus with LNM. Neutrophils could be detected in about half of lymphatic cancer emboli in T1b tumors, namely lymphatic tumor cell-neutrophil clusters. The prevalence of neutrophils in lymphatic cancer embolus was 46.97% in T1b tumors and 45.61% in SM2 tumors, respectively (**Figure 1I**, **Table s2D**). Incidence of LNM in patients with neutrophil-free cancer emboli was 42.86% in T1b tumors and 41.94% in SM2 tumors, whereas it was increased to 77.42% (*P* = 0.006) in T1b tumors (**Figure 1J**) and 80.77% (*P* = 0.003) in SM2 tumors (**Figure 1K**) with lymphatic tumor cell-neutrophil clusters, respectively, suggesting that presence of lymphatic tumor cell-neutrophil clusters is prone to LNM. According to numbers of neutrophils in embolus, the emboli were divided into three groups, high- (> 10 neutrophils *per* embolus), moderate- (1~10 neutrophils *per* embolus) and few-neutrophil group (< 1 neutrophil *per* embolus). Intriguingly, the abundance of TANs in tumors was positively associated with neutrophils in lymphatic cancer emboli (*P* = 0.001 in T1b tumors and SM2 tumors, **Table s2E**), suggesting that neutrophils in emboli originate from TANs. However, the number of neutrophils in emboli had no influence on LNM (**Table s2D**,** Figure 1J**, **1K**), which indicates that neutrophils in lymphatic cancer emboli promote LNM, regardless of its number.

Taken together, these clinicopathologic studies indicate that TANs promote lymphatic invasion and LNM, namely enhancing tumor invasiveness in gastric cancer.

### Interaction between neutrophils and tumor cells promotes tumor EMT and enhances tumor cell invasiveness

**Figure 2:**
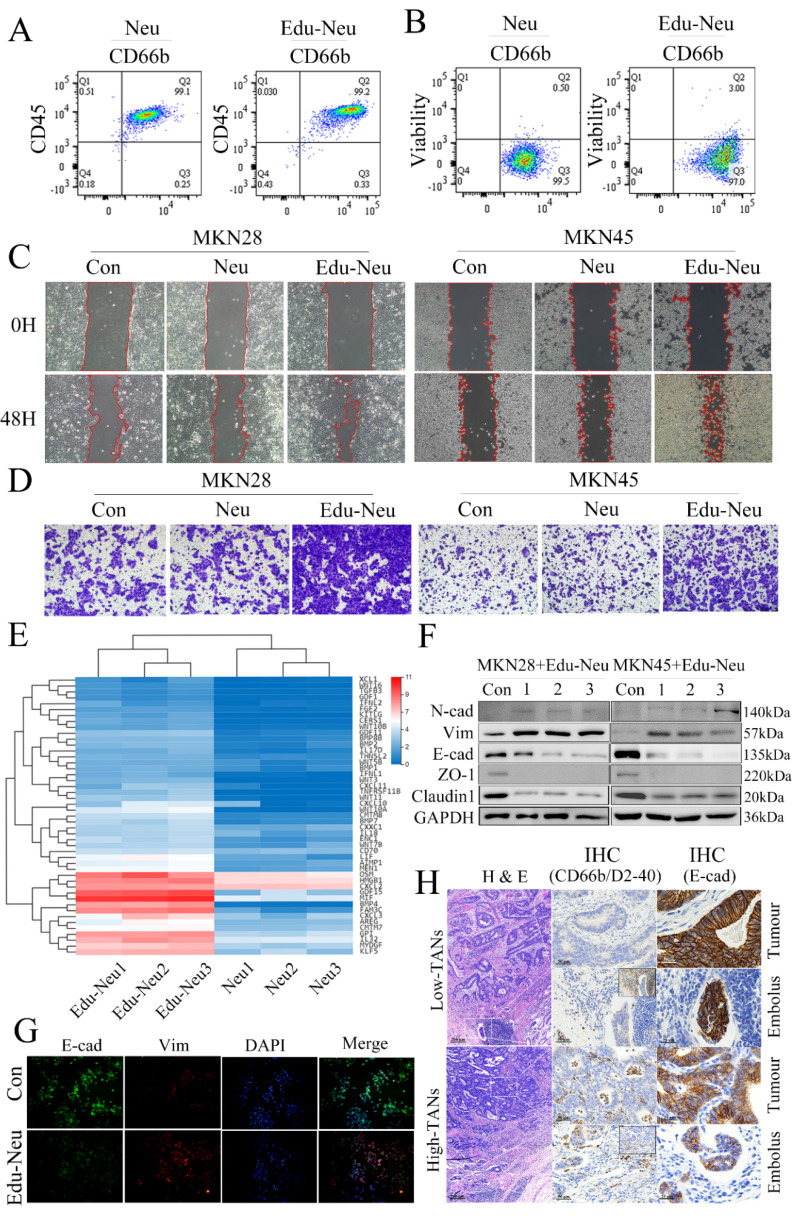
** Interaction between neutrophils and tumor cells promotes tumor EMT and enhances tumor cell invasion.** (**A**, **B**) FCM analyses showed that co-culture with MKN45 cells exerted no remarkable influence on purity and viability of isolated neutrophils. (**C**, **D**) After co-culture with MKN28 or MKN45 cells, the CMs of Edu-Neus were used to treat fresh MKN28 or MKN45 cells, and cell migration (**C**) and invasion (**D**) were increased significantly (con, MKN28 or MKN45 cells in complete RPMI1640; Neu, MKN28 or MKN45 cells treated with CMs of isolated neutrophils; Edu-Neu, MKN28 or MKN45 cells treated with CMs of educated-neutrophils) (×100). (**E**) RNA-seq showed forty-seven differential expressed genes among “cytokine activity” category in “molecular function” after co-culture with MKN45, and the four most up-regulated genes were GDF15, MIF, BMP4 and FAM3C. (**F**) Isolated peripheral neutrophils from healthy donors (1, 2 and 3) were co-cultured with MKN28 or MKN45 cells, respectively. Subsequently, these neutrophils were harvested, and their CMs were applied to treat fresh MKN28 or MKN45 cells. The expression of E-cad, ZO-1 and Claudin-1 was decreased, while the expression of Vim and N-cadherin (N-cad) was increased. (**G**) Immunofluorescence assays showed that treatment with the Edu-Neu's CM increased Vim expression, and decreased E-cad expression (×400). (**H**) The left lane showed H & E images of tumor tissues of high-TANs group and low-TANs group. The middle lane showed IHC images indicating presence of TANs and lymphatic vessels with CD66b and D2-40 (right-upper frame), respectively. The right lane showed E-cad expression in tumor tissues or lymphatic cancer emboli. E-cad expression in tumor tissues or lymphatic cancer emboli was decreased in high-TANs group compared with that in low-TNAs group, and E-cad levels in lymphatic cancer emboli were further decreased with regard to tumor tissues in high-TANs group. (Neu, neutrophil; Edu-Neu, tumor-educated neutrophil).

To investigate the molecular mechanism underlying TANs-facilitated tumor invasion in gastric carcinoma, the effects of neutrophils on tumor cell migration and invasion were assayed. First, the peripheral neutrophils from healthy donors or patients with locally advanced gastric cancer were isolated (**Figure s1A**) and cultured for certain time to generate CMs. Human stomach cancer cell lines, MKN28 and MKN45, were further treated with these CMs respectively. These CMs, either from healthy donors or gastric cancer patients, had no impact on tumor cell migration and invasion (**Figure s1B**, **s1C**, **s1D**, **s1E**). Then, the isolated neutrophils from healthy donors were co-cultured with MKN28 or MKN45 cells for 6 h. FCM analyses showed that this co-culture exerted no remarkable influence on purity and viability of neutrophils (**Figure 2A**, **2B**, **s1F**, **s1G**). Next, the tumor-treated neutrophils were collected and purified. After additional 12-hour culture, the CMs were collected. Notably, the CMs from tumor-treated neutrophils significantly promoted migration and invasion of MKN28 or MKN45 cells (**Figure 2C**, **2D**, **s1H**, **s1I**). These results clearly suggest that interaction between neutrophils and tumor cells, namely educated-neutrophils (Edu-Neus) by tumor cells, is required for neutrophils-mediated tumor cell invasiveness.

To elucidate how these Edu-Neus enhance tumor cell invasiveness, RNA sequencing (RNA-seq) was performed to analyze the gene profiles of peripheral neutrophils from three healthy donors with or without co-culture with MKN45 cells for 6 h. Multiple differentially expressed genes (DEGs) were identified from MKN45-educated neutrophils, including (7457, 7440, 7646) up-regulated genes and (1378, 1623, 2190) down-regulated genes, respectively (**Figure s2A**). 7531 DEGs overlapped in three donors, including 6698 up-regulated genes and 833 down-regulated genes (**Figure s2B**). According to GO analysis, the 7531 DEGs were enriched in 62 terms, including 30 in “biological process”, 18 in “cell component”, and 14 in “molecular function” categories. The GO term enrichment analyses revealed that mitochondrial translation, mitochondrial translational elongation, and cytoplasmic translation are the three most significantly enriched terms within “biological process”; small ribosomal subunit and cytosolic large ribosomal subunit are most enriched in “cell component”; and structural constituent of ribosome, snoRNA binding and rRNA binding are most enriched in “molecular function” categories (**Figure s2C**, **s2D**). Subsequently, KEGG pathway analysis indicated that these DGEs were abundant in signaling pathways involved in cell growth and death, signal transduction, transcription, cancer, and immune system. These DEGs were mainly enriched in the regulations for ribosome, RNA transport, cell cycle and aminoacyl-tRNA biosynthesis (**Figure s2C**, **s2D**). These results indicate that co-culture with gastric cancer cell leads to far-reaching molecular alterations in neutrophils, which may be involved in the development of TANs within the gastric cancer TME.

TANs were proposed to function on tumor cells by producing certain cytokines. Forty-seven genes were obtained among the “cytokine activity” category in “molecular function” meeting Log2 (FC) > 1 and false discovery rate (FDR) < 0.05 (**Figure 2E**). The four most up-regulated genes in the Edu-Neus were growth differentiation factor 15 (GDF15), macrophage migration inhibitory factor (MIF), bone morphogenetic protein 4 (BMP4) and FAM3C. These genes have been reported to be involved in tumor EMT among various cancer types 30-34, and EMT is associated with multiple tumor cell functions, including invasiveness 21, 22. Thus, we speculated that the Edu-Neus can promote tumor invasion *via* inducing EMT. MKN28 and MKN45 cells were treated with CMs derived from neutrophils of healthy donors or gastric cancer patients, respectively. Similar to aforementioned assays, these CMs exerted no effect on EMT marker expression of tumor cells (**Figure s3A**, **s3B**). We then switched to utilize CMs from Edu-Neus to treat MKN28 or MKN45 cells, respectively. Interestingly, E-cad expression was inhibited whereas Vim was increased in these tumor cells after treatment with CMs (**Figure 2F**, **2G**, **s3C**). These results suggest that Edu-Neus promote tumor cell EMT, which in turn enhance tumor cell invasiveness. Of note, crosstalk of neutrophils with tumor cells is prerequisite in this process. Next, the EMT status of gastric cancer specimen and its correlation with TAN abundance were evaluated. E-cad expression in tumor tissues or lymphatic cancer emboli was decreased in high-TANs group compared with that in the low-TNAs group. Furthermore, E-cad expression levels in lymphatic cancer emboli were even further decreased with regard to tumor tissues in high-TANs group (**Figure 2H**, **s3D**).

### FAM3C is involved in tumor cell EMT induced by Edu-Neus

**Figure 3:**
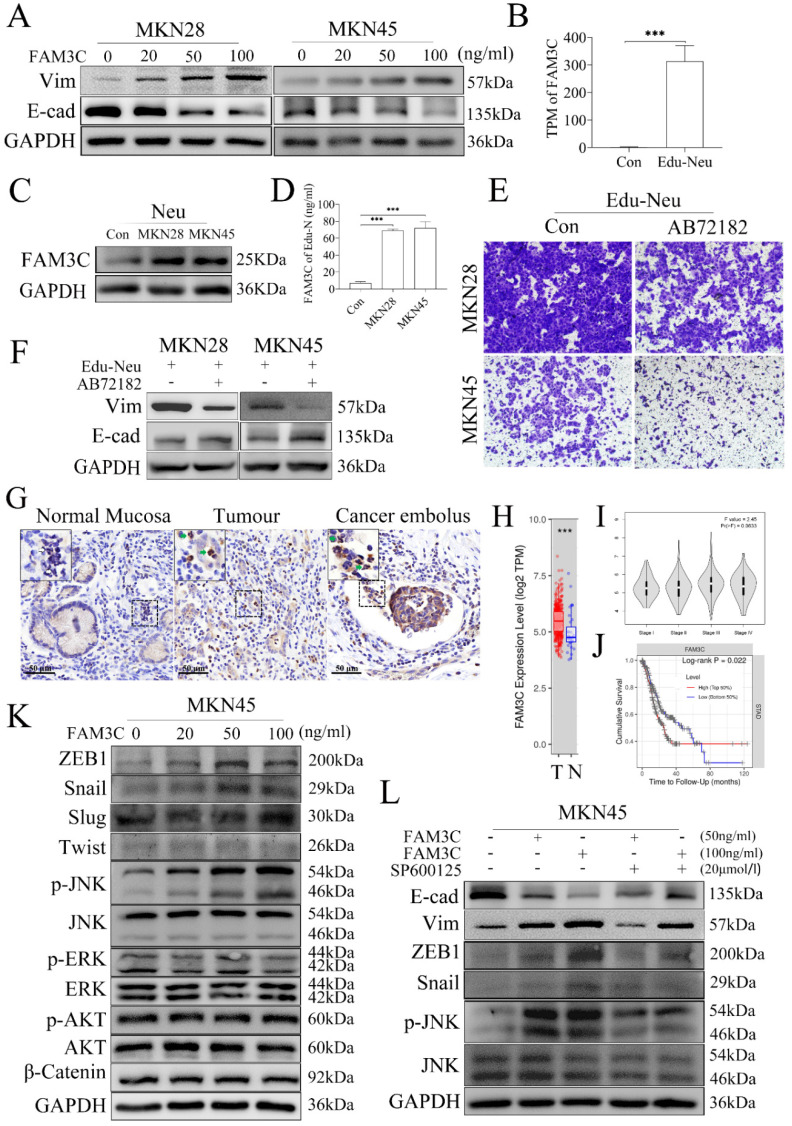
** FAM3C is involved in tumor cell EMT induced by Edu-Neus.** (**A**) FAM3C treatment decreased E-cad expression and increased Vim expression in MKN28 and MKN45 cells in a dose-dependent manner. (**B**) RNA-seq analysis showed FAM3C mRNA was increased by more than 140 times in Edu-Neus. (**C**, **D**) Co-culture with MKN28 or MKN45 cells increased FAM3C level (**C**) or production (**D**) in neutrophils. (**E**, **F**) Blockage of FAM3C with AB72182 reversed the enhanced-invasiveness (**E**, ×100) or induced-EMT (**F**) of tumor cells mediated by Edu-Neus. (**G**) FAM3C immunostaining was detected in TANs in human gastric tumor tissues and cancer emboli with IHC (green arrow), but it was not detected in neutrophils in normal stomach tissues (blank arrow). (**H**) FAM3C mRNA levels in gastric tumor tissues (T) were much higher than that in corresponding normal stomach tissues (N). (**I**) FAM3C level in gastric tumors was related to TNM staging of gastric cancer although it was not significant (*P* = 0.063). (**J**) FAM3C level in tumors was negatively associated with cumulative survival of gastric cancer patients (*P* = 0.022). (**K**) FAM3C treatment up-regulated p-JNK as well as ZEB1 and Snail expression in MKN45 cells in a dose-dependent manner but exerted no marked effects on expression of p-ERK, p-Akt, Slug, and β-Catenin. (**L**) The expressions of E-cad, Vim and ZEB1 or Snail were reversed with JNK inhibitor treatment. (***, *P* < 0.001) (Neu, neutrophil; Edu-Neu, tumor-educated neutrophil).

To identify which factor is responsible for tumor EMT induced by Edu-Neus, we found that FAM3C could decrease E-cad expression and increase Vim expression in MKN28 or MKN45 cells with a dose-dependent manner (**Figure 3A**,** s4A**). However, GDF15, MIF and BMP4 had no similar effects (**Figure s4B**, **s4C**, **s4D**). Notably, FAM3C gene expression was increased by more than 140 times in the RNA-seq analysis (**Figure 3B**), which was further confirmed by assays of western blotting and ELISA (**Figure 3C**,** 3D**,** s4E**). Furthermore, blockage of FAM3C with a neutralizing antibody, AB72182, could reverse the enhanced-invasiveness and induced-EMT of tumor cells mediated by Edu-Neus (**Figure 3E**, **3F**, **s4F**, **s4G)**. Importantly, FAM3C was strongly expressed in TANs in human gastric cancer tissues and cancer emboli with IHC assays (**Figure 3G**,** s4H**).

Bioinformatics analysis using the data of stomach cancer patients from the Cancer Genome Atlas (TCGA) revealed that FAM3C mRNA level in gastric cancer tissues was much higher than that in the corresponding normal stomach tissues (**Figure 3H**, *P* < 0.001). Furthermore, FAM3C level in tumors was related to TNM staging of gastric cancer although it was not significant (**Figure 3I**, *P* = 0.063), and FAM3C level in tumors was inversely associated with cumulative survival of stomach cancer patients (**Figure 3J**, *P* = 0.022).

To dissect which signaling is regulated by FAM3C and involved in tumor EMT mediated by Edu-Neus, we found that FAM3C treatment up-regulated the expression of phosphorylated JNK (p-JNK) and EMT-associated transcription factors, ZEB1 and Snail, in MKN45 cells in a dose-dependent manner, but exerted no marked effects on expression of p-ERK, p-Akt, Slug and β-Catenin (**Figure 3K**, **s4I**). After treatment with SP600125, an inhibitor for JNK signaling, the expression of E-cad, Vim, ZEB1 and Snail mediated by FAM3C was reversed, suggesting that FAM3C promotes tumor cell EMT *via* JNK-ZEB1/Snail signaling activation (**Figure 3L**,** s4J**).

### Gastric cancer cells educate neutrophils via TGFβ1

**Figure 4:**
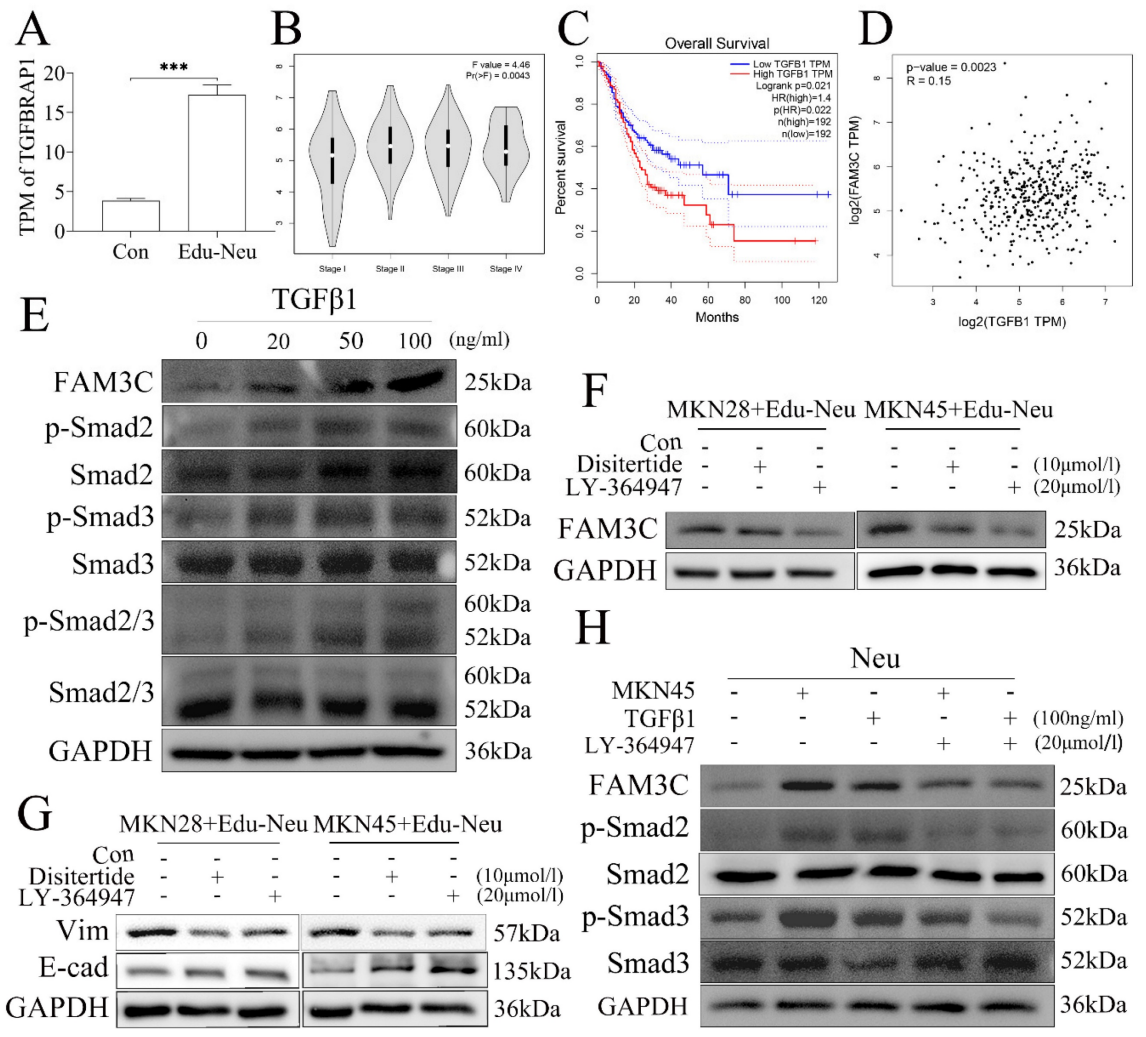
** Gastric cancer cells educate neutrophils *via* TGFβ1.** (**A**) TGFBRAP1 expression was increased in Edu-Neus (*P* = 0.000). (**B**) TGFβ1 mRNA level in tumor tissues was positively associated with TNM staging (*P* = 0.004) and overall survival negatively (**C**) (*P* = 0.022). (**D**) TGFβ1 mRNA level was correlated with FAM3C level in gastric cancer (*P* = 0.002). (**E**) Exogenous TGFβ1 up-regulated FAM3C expression in neutrophils in a dose-dependent manner, and the expressions of p-Smad2, p-Smad3 or p-Smad2/3 were up-regulated concurrently. (**F**) Treatment with TGFβ1 inhibitor, Disitertide, or TGFβ1 receptor (TGFβ1R) inhibitor, LY-364947, inhibited FAM3C expression. (**G**) Treatment with Disitertide or LY-364947 reversed expression of EMT markers in tumor cells. (**H**) LY-364947 down-regulated FAM3C and p-Smad2 or p-Smad3 expression in neutrophils simultaneously. (Neu, neutrophil; Edu-Neu, tumor-educated neutrophil)

We next investigated the potential molecular signaling responsible for tumor cell-mediated up-regulation of FAM3C in neutrophils during their crosstalk. The RNA-seq data analysis showed that transforming growth factor beta receptor associated protein 1 (TGFBRAP1) gene expression was increased in Edu-Neus (**Figure 4A**, *P* = 0.000). Therefore, we proposed that tumor cells-derived transforming growth factor-β1 (TGFβ1) may be involved in FAM3C up-regulation in neutrophils. Bioinformatics analysis with TCGA data revealed that TGFβ1 mRNA level in stomach cancer specimen was positively correlated with TNM stage (**Figure 4B**, *P* = 0.004), but negatively associated with overall survival (**Figure 4C**, *P* = 0.022). Furthermore, TGFβ1 mRNA level was positively correlated with FAM3C level in gastric cancer (**Figure 4D**, *P* = 0.002). Exogenous TGFβ1 could up-regulate FAM3C expression in neutrophils in a dose-dependent manner (**Figure 4E**, **s5A**). In addition, TGFβ1 treatment concurrently up-regulated phosphorylated Smad2 (p-Smad2), p-Smad3 or p-Smad2/3 in neutrophils in a dose-dependent manner (**Figure 4E**,** s5A**).

Furthermore, treatment with TGFβ1 inhibitor, Disitertide, or TGFβ1 receptor inhibitor, LY-364947, significantly inhibited FAM3C expression (**Figure 4F**,** s5B**) in neutrophils, and reversed expression of E-cad and Vim in tumor cells (**Figure 4G**,** s5C**), respectively. Treatment with LY-364947 could also down-regulate FAM3C and p-Smad2 or p-Smad3 expression in neutrophils simultaneously (**Figure 4H**,** s5D**). Collectively, these results strongly suggest that tumor cells increase FAM3C levels through TGFβ1-Smad2/3 pathway in neutrophils during their interaction, and FAM3C-mediated tumor cell EMT is through JNK-ZEB1/Snail signaling activation. This detrimental loop enhances tumor cell invasiveness and conceivably promotes LNM in gastric cancer.

### Crosstalk with tumor cells enhances the affinity of neutrophils with tumor cells through interaction between integrins α6β1 and α6β4 with CD151

**Figure 5:**
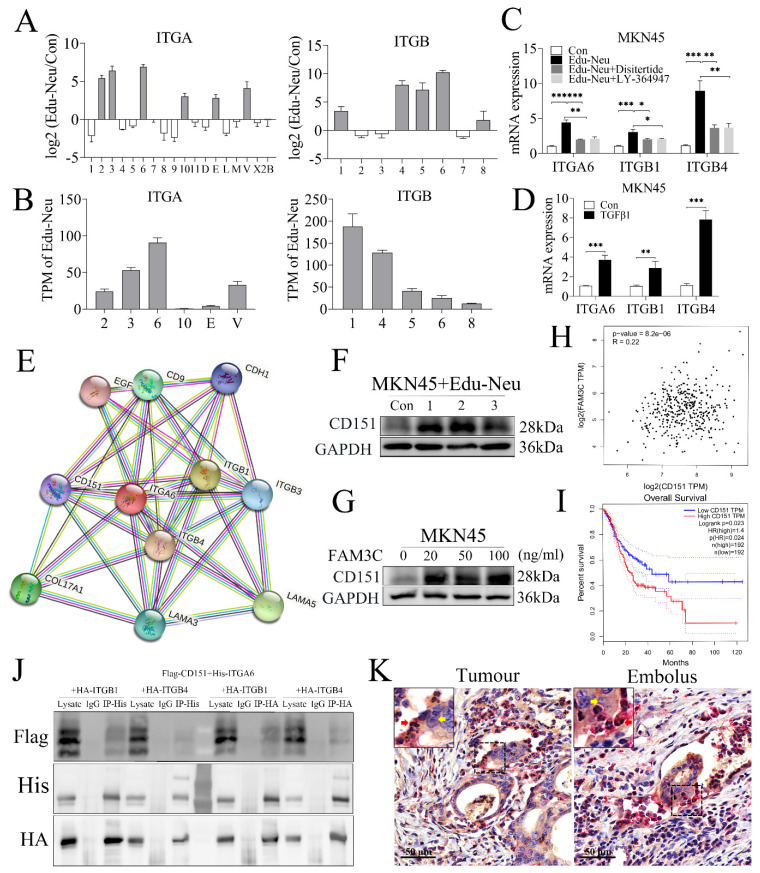
** Crosstalk with tumor cells enhances the affinity of neutrophils with tumor cells through interaction between integrins α6β1 and α6β4 with CD151.** (**A**) RNA-seq analysis showed that several integrins, including α2, α3, α6, α10, αE, αv, β1, β4, β5, β6 and β8, were up-regulated in Edu-Neus. (**B**) The subunits α6 (ITGA6), β1 (ITGB1) and β4 (ITGB4) were the most significantly increased ones. (**C**) Co-culture with MKN45 cells up-regulated expression of the three subunits by qPCR assays, and treatment with Disitertide or LY-364947 could attenuate these effects. (**D**) Exogenous TGFβ1 increased the levels of these three subunits in neutrophils. (**E**) Prediction analysis of protein-protein interaction showed that ITGA6, ITGB1 and ITGB4 can interact with CD151. (**F**) Co-culture with neutrophils could increase CD151 expression in MKN45 cells. (**G**) FAM3C treatment could increase CD151 expression in MKN45 cells. (**H**) CD151 mRNA levels were correlated positively with FAM3C levels in gastric tumor tissues. (**I**) CD151 mRNA level in gastric tumor tissues was negatively associated with overall survival of gastric cancer patients. (**J**) Co-IP assays showed that ITGA6, ITGB1 and ITGB4 could bind CD151. (**K**) Subunit α6 was detected in neutrophils (red arrow, red) and CD151 was found in tumor cells (yellow arrow, yellow) using dual-color IHC assays for lymphatic cancer emboli. (*, *P* < 0.05; **, *P* < 0.01; ***, *P* < 0.001) (Edu-Neu, tumor-educated neutrophil).

The aforementioned clinicopathologic analyses clearly showed that lymphatic tumor cell-neutrophil clusters were associated with tumor LNM. However, it still remains unclear how the clusters form. The RNA-seq analysis has shown that several integrins, including α2, α3, α6, α10, αE, αv, β1, β4, β5, β6 and β8, were increased in Edu-Neus (**Figure 5A**), and the subunits α6 (ITGA6), β1 (ITGB1) and β4 (ITGB4) were the most significant ones (**Figure 5B**). Co-culture with MKN45 cells up-regulated gene expression of these three subunits, and treatment with Disitertide or LY-364947 could attenuate the effects (**Figure 5C**). Furthermore, exogenous TGFβ1 also increased the levels of these three subunits in neutrophils (**Figure 5D**), suggesting that tumor cells up-regulate ITGA6, ITGB1 and ITGB4 in neutrophils through TGFβ1 regulation. Thus, it was reasoned that interaction of neutrophils *via* integrins with tumor cells is involved in the formation of lymphatic tumor cell-neutrophil clusters. Prediction analysis of protein-protein interaction (https://www.string-db.org/cgi/network?taskId=bNu4rqFjQNrM&sessionId=bWYYYI4kyuG1) indicated that the subunits α6, β1 and β4 can interact with CD151 (**Figure 5E**). As expected, co-culture with neutrophils or FAM3C treatment increased CD151 levels in MKN45 cells (**Figure 5F**, **5G**, **s5E**, **s5F**). Bioinformatics analysis with TCGA data indicated that CD151 mRNA level was positively correlated with FAM3C level in gastric cancer (**Figure 5H**) and was inversely associated with overall survival of stomach cancer patients (**Figure 5I**, *P* = 0.024). To determine the physical interactions between integrins α6β1 and α6β4 with CD151, co-IP assays showed that α6, β1 or β4 subsets could bind CD151 (**Figure 5J**). Furthermore, subunit α6 was detected in neutrophils and CD151 was observed in tumor cells using the dual-color IHC assays for lymphatic cancer emboli (**Figure 5K**). Collectively, our results suggest that integrins α6β1 and α6β4 in TANs expedite tumor invasion *via* interaction with CD151 in tumor cells, which results in the formation of lymphatic tumor cell-neutrophil clusters.

### Neutrophil depletion prevents gastric cancer cell tumorigenesis through inhibiting tumor EMT in the gastric tumor model

**Figure 6:**
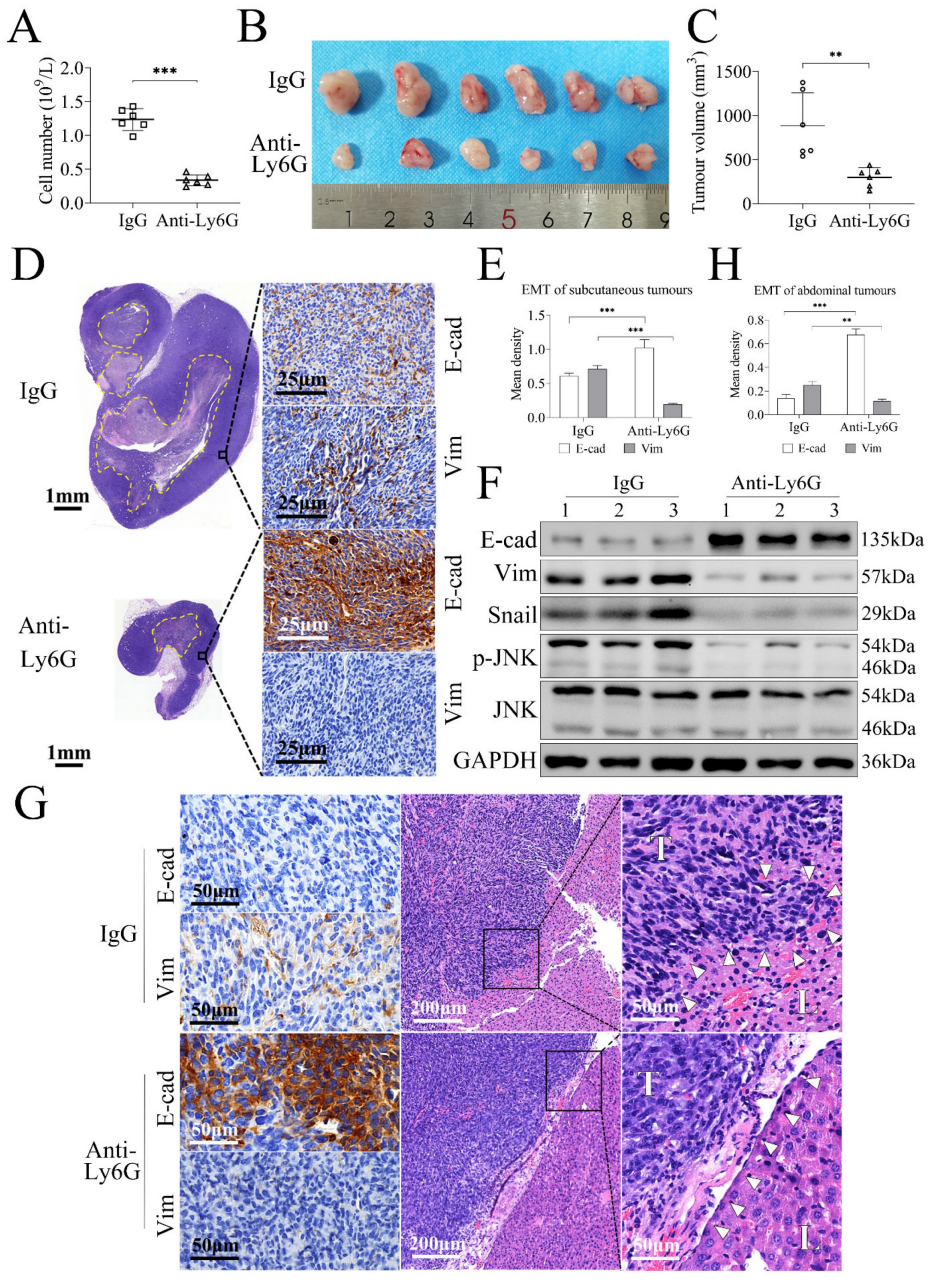
** Neutrophil depletion prevents gastric cancer cell tumorigenesis through inhibiting tumor EMT in tumor-bearing mice.** (**A**) Treatment with anti-Ly6G antibody decreased the numbers of peripheral neutrophils significantly compared with control IgG treatment (*P* = 0.000). (**B**, **C**) The subcutaneous allograft models study showed that tumor volumes in anti-Ly6G-treated group were smaller than that in IgG-treated group. (**D**, **E**, **F**) Further assays revealed that E-cad level was increased whereas expressions of Vim, Snail and p-JNK were decreased significantly in anti-Ly6G-treated group with relative to IgG-treated group (dotted line, necrotic area in tumor). (**G**, **H**) The abdominal allograft models also showed E-cad was up-regulated while Vim was down-regulated in anti-Ly6G-treated group compared with IgG-treated group. Tumor cells were spindle in IgG-treated group, and tumors invaded into liver and the liver envelope structure was destroyed (white triangular arrow), whereas tumor cells were polygonal in anti-Ly6G-treated group, and tumor was not found to invade into liver and the liver envelope was intact (white triangular arrow). (**, *P* < 0.01; ***, *P* < 0.001).

The allograft tumor models of gastric cancer in C57BL/6 mice using MFC cells, a mouse stomach cancer cell line, were established to investigate the effects of neutrophil depletion on tumorigenesis and tumor invasion. Treatment with anti-Ly6G could decrease the numbers of peripheral neutrophils significantly compared with the control IgG treatment (*P* = 0.000, **Figure 6A**). The subcutaneous tumor model showed that the tumor volumes in anti-Ly6G-treated group (239.10 ± 108.35 mm^3^) were smaller than those in IgG-treated group (785.16 ± 372.17 mm^3^) (*P* = 0.004, **Figure 6B**, **6C**). Further assays revealed that E-cad was increased whereas expressions of Vim, Snail, and p-JNK were decreased significantly in anti-Ly6G-treated group with relative to the IgG-treated group (**Figure 6D**, **6E**, **6F**, **s5G**), suggesting that neutrophil depletion prevents gastric cancer tumorigenesis through inhibiting EMT.

The abdominal model further approved the changing modes of E-cad and Vim expression in tumor tissues (**Figure 6G**, **6H**), but the difference of tumor focus numbers or tumor burden was not appropriate to be evaluated. Pathologically, tumor cells were spindle in IgG-treated group, and tumors invaded into liver and liver envelope structure was destroyed, suggesting that MFC cells have strong invasiveness. However, in anti-Ly6G-treated group, tumor cells were polygonal, and tumor invasion into liver was not observed and liver envelope was intact (**Figure 6G**), suggesting that neutrophil depletion inhibits MFC cell invasion. Collectively, these findings indicate that neutrophils promote tumor invasion through mediating EMT.

## Discussion

Herein, we further demonstrated that TANs enhance tumor invasiveness in gastric cancer. Crosstalk between neutrophils and tumor cells is required for enhanced tumor invasiveness, and confers neutrophils with properties to promote tumor cell EMT, which in turn promotes tumor invasion. Mechanistically, co-culture with tumor cells increases FAM3C levels in neutrophils regulated by TGFβ1-Smad2/3 signaling activation, and FAM3C promotes tumor cell EMT through JNK-ZEB1/Snail pathway. This crosstalk enhanced the affinity of neutrophils with tumor cells through interaction of integrins α6β1 and α6β4 with CD151. Studies with tumor-bearing mice further demonstrated neutrophils can enhance gastric cancer cell tumorigenesis and invasiveness through promoting tumor EMT. The novel studies identify the accomplice roles of TNAs in promoting gastric cancer invasion, and provide potential strategies in prevention and/or treatment of LNM in gastric carcinoma and other cancers as well.

Our previous work and other studies have demonstrated that high levels of TANs are positively associated with frequency of LNM and can predict dismal prognosis in gastric cancer 19, 20. This study further suggests that T1b gastric cancer has certain incidence of LNM compared with T1a tumors, making it suitable for probing the mechanisms of LNM. Our comprehensive clinicopathologic investigations have demonstrated that accumulated TANs promote LNM in T1b tumors. Lymphatic invasion is the key step for LNM. We further revealed that high levels of TANs serve as the independent risk factor for lymphatic invasion, and that lymphatic tumor cell-neutrophil clusters are positively associated with high frequency of LNM.

Further studies have shown that peripheral neutrophils from healthy donors enhance tumor cell invasiveness after co-culture with gastric cancer cells. But the mere peripheral neutrophils, even from gastric cancer patients, did not have similar effects, which indicated that the interaction between neutrophils and cancer cells is prerequisite for the neutrophil transformation. These results suggested that TANs are educated by tumor cells to obtain these distinct abilities 9, 35. Furthermore, RNA-seq analysis showed that co-culture with tumor cells induces extensive gene expression alterations in neutrophils, which suggests that these neutrophils are educated to transform into active state. Multiple mitochondrial-related genes were up-regulated in Edu-Neus, which are involved in aminoacyl-tRNA biosynthesis and Ribosome. These results indicated that protein synthesis function of neutrophils was activated by interaction with tumor cells, and Edu-Neus can synthesize more proteins, including cytokines, to promote tumor progression including EMT. All the four most DEGs, GDF15, MIF, BMP4 and FAM3C, are the transcription factors for EMT, suggesting that Edu-Neus obtain the pro-EMT signature. The crosstalk between neutrophils and tumor cells promotes tumor cell EMT, which was confirmed by assays with human tumor specimens. EMT has been demonstrated to enhance motility and invasiveness of cancer cells, which in turn promotes lymphatic vessel intravasation 6, 10. Following the Th1/Th2 paradigm, TANs have been found to exhibit N1 (tumor-suppressive) or N2 (tumor-promoting) phenotype 9, 14, 36. Based on the gene expression alterations (**Table s3**) and the pro-EMT activities, Edu-Neus were enabled to obtain N2 phenotype. It is established that EMT is a binary process with two distinct cell populations, epithelial and mesenchymal. However, EMT has been shown to occur in a gradual way characterized by several cellular states expressing varied levels of epithelial and mesenchymal markers. The transitional states between epithelial and fully mesenchymal states are referred as partial or hybrid EMT states 21, 37. Our studies showed that E-cad levels in lymphatic cancer emboli was much lower than that in primary tumor tissues in high-TANs group, suggesting that TANs-mediated EMT is a hybrid state with high metastatic potential.

Mechanistically, we identified that only FAM3C is involved in EMT mediated by Edu-Neus. As a cytokine-like gene, FAM3 family includes FAM3A, FAM3B, FAM3C, and FAM3D, and is involved in the occurrence and development of several cancers. In the present study, only FAM3A and FAM3C were detected in Edu-Neus, and FAM3C was increased significantly while FAM3A was up-regulated slightly (data not shown). FAM3C contributes to EMT 31, 32, 38, 39, and has been revealed to be up-regulated in many cancer types 38. However, we found that FAM3C is prominently expressed in neutrophils in gastric tumor tissues and is detected weakly in tumor cells. Our present study and several other reports 38, 40 showed that FAM3C levels are positively correlated with poor prognosis in cancer patients, making FAM3C as a novel biomarker or potential therapeutic target for cancer. We further identified that FAM3C promotes gastric cancer cell EMT *via* JNK-ZEB1/Snail signaling activation, instead of Akt pathway, which was referred by other studies 41, 42. Furthermore, we demonstrated that FAM3C up-regulation in neutrophils is through tumor-derived TGFβ1. TGFβ1 commonly leads to a partial EMT in cancer cells, and these EMT-related changes promote cancer cell migration and invasion, which are preconditions for cancer dissemination 43, 44. The crosstalk between gastric cancer cells and TANs clearly suggests that, in addition to exerting on tumor cells by autocrine way, tumor cell-derived TGFβ1 can promote tumor EMT by educating neutrophils in gastric cancer TME.

Tumor cell-neutrophil clusters have been reported to be mainly found in bloodstream and are highly efficient in initiating metastasis, and their presence is associated with poor prognosis in cancer patients 45, 46. Our study identified, for the first time, the presence of tumor cell-neutrophil clusters in lymphatic vessels in gastric cancer, which is prone to LNM. Notably, we revealed that integrins α6β1 and α6β4 in Edu-Neus can interact with CD151 in stomach cancer cells, which results in lymphatic tumor cell-neutrophil cluster formation and facilitates tumor invasion.

Furthermore, our allograft tumor model study showed that neutrophil depletion suppresses gastric cancer cell tumorigenesis and invasiveness through inhibiting EMT. The preliminary result suggests that targeting TANs might be a feasible therapeutic strategy for gastric carcinoma. Various methods targeting TANs have achieved a promising response and prevent cancer relapse 47, 48. Due to the phenotypic and functional diversity of TANs, targeting specific TAN functions in the TME may generate translatable therapeutic approaches 16. Our studies uncovered the functional roles of TANs in tumor invasion and LNM in gastric cancer. However, tumor LNM involves multiple steps, the precise roles of TANs in lymphangiogenesis, cancer cell extravasation, cancer cell migration in lymphatic vessels and TDLN immunoediting remain to be investigated.

**Figure 7:**
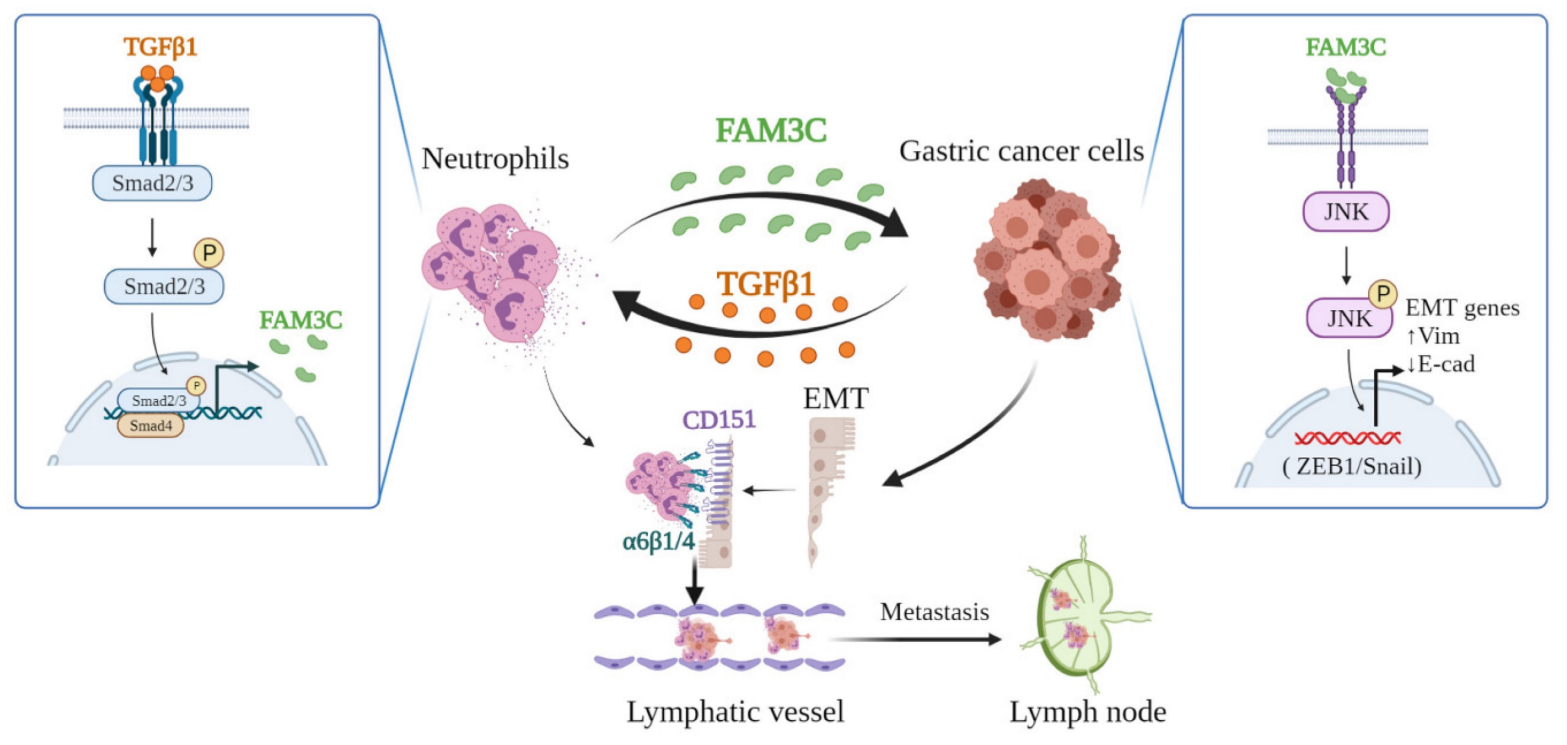
Depiction of mechanism underlying TANs-mediated tumor invasiveness and LNM.

In conclusion, our study demonstrates that tumor cells educate TANs *via* TGFβ1 to produce more FAM3C, which mediates tumor cell EMT. This detrimental loop enhances tumor cell invasiveness, facilitates formation of lymphatic tumor cell-neutrophil clusters, and promotes LNM (**Figure 7**), which indicates that targeting TANs is a potential strategy to improve the prognosis of stomach carcinoma.

## Supplementary Material

Supplementary figures and tables.

## Figures and Tables

**Table 1:** Clinical relevance of TANs in T1b gastric cancer.

Clinicopathologic Features		TANs		χ^2^	*P*
		Low (n=137) (%)	High (n=120) (%)		
Gender	Male	97 (53.0)	86 (47.0)	0.023	0.891
	Female	40 (54.1)	34 (45.9)		
Age (year)	<65	76 (55.9)	60 (44.1)	0.565	0.384
	≥65	61 (50.4)	60 (49.6)		
Tumor location in the stomach	Upper third	36 (55.4)	29 (44.6)	0.170	0.934
	Middle third	35 (52.2)	32 (47.8)		
	Lower third	66 (52.8)	59 (47.2)		
Tumor diameter (cm)	<2	55 (51.9)	51 (48.1)	0.365	0.854
	2~3	47 (56.0)	37 (44.0)		
	≥3	35 (52.2)	32 (47.8)		
Macroscopic type	Elevated	9 (31.0)	20 (69.0)	7.115	0.026
	Flat	13 (65.0)	7 (35.0)		
	Depressed	115 (55.3)	93 (44.7)		
Depth of invasion	Sm1	38 (55.1)	31 (44.9)	0.041	0.779
	Sm2	99 (52.7)	89 (47.3)		
Lauren classification	Intestinal	93 (57.1)	70 (42.9)	9.551	0.022
	Diffuse	19 (67.9)	9 (32.1)		
	Mixed	19 (38.8)	30 (61.2)		
	Not defined	6 (35.3)	11 (64.7)		
Histological classification	Well	31 (75.6)	10 (24.4)	10.798	0.004
	Moderately	61 (46.6)	70 (53.4)		
	Poorly	45 (52.9)	40 (47.1)		
Lymphatic invasion	Absence	114 (59.7)	77 (40.3)	11.179	0.001
	Presence	23 (34.8)	43 (65.2)		
Perineural invasion	Absence	129 (53.5)	112 (46.5)	0.000	0.802
	Presence	8 (50.0)	8 (50.0)		
*H. pylori* infection	Absence	101 (54.3)	85 (45.7)	0.142	0.675
	Presence	36 (50.7)	35 (49.3)		
Lymph node metastasis	Absence	120 (61.2)	76 (38.8)	19.476	0.000
	Presence	17 (27.9)	44 (72.1)		

## Abbreviations

LNM: lymph node metastasis
TANs: tumor-associated neutrophils
EGC: early gastric cancer
RNA-seq: RNA sequencing
co-IP: co-immunoprecipitation
EMT: epithelial-to-mesenchymal transition
TDLNs: tumor-draining lymph nodes
TME: tumor microenvironment
ACRG: Asian Cancer Research Group
MSI: microsatellite instability
MSS: microsatellite stability
H & E: hematoxylin & eosin
IHC: immunohistochemistry
E-cad: E-cadherin
Vim: vimentin
CM: conditioned medium
Edu-Neu: tumor-educated neutrophils
DEGs: differential expressed genes
GO: Gene Ontology
KEGG: Kyoto Encyclopedia of Genes and Genomes annotations
qPCR: real-time quantitative PCR
GDF15: growth differentiation factor 15
MIF: macrophage migration inhibitory factor
BMP4: bone morphogenetic protein 4
FAM3C: family with sequence similarity 3 member C
TCGA: the Cancer Genome Atlas
p-JNK: phosphorylated JNK
TGFBRAP1: transforming growth factor beta receptor associated protein 1
TGFβ1: transforming growth factor-β1
TGFβ1R: TGFβ1 receptor
ILEI: interleukin-like EMT inducer
LIFR: leukemia inhibitory factor receptor
