# Supplementary material for: Neutrophils promote tumor invasion via FAM3C-mediated epithelial-to-mesenchymal transition in gastric cancer
Source: Int J Biol Sci. 2023 Feb 21;19(5):1352–68. doi: 10.7150/ijbs.79022 (PMC10086748; doi:10.7150/ijbs.79022)
Supplement: Supplementary file 1 — Supplementary figures and tables. [file ijbsv19p1352s1.pdf]

Supplementary Materials

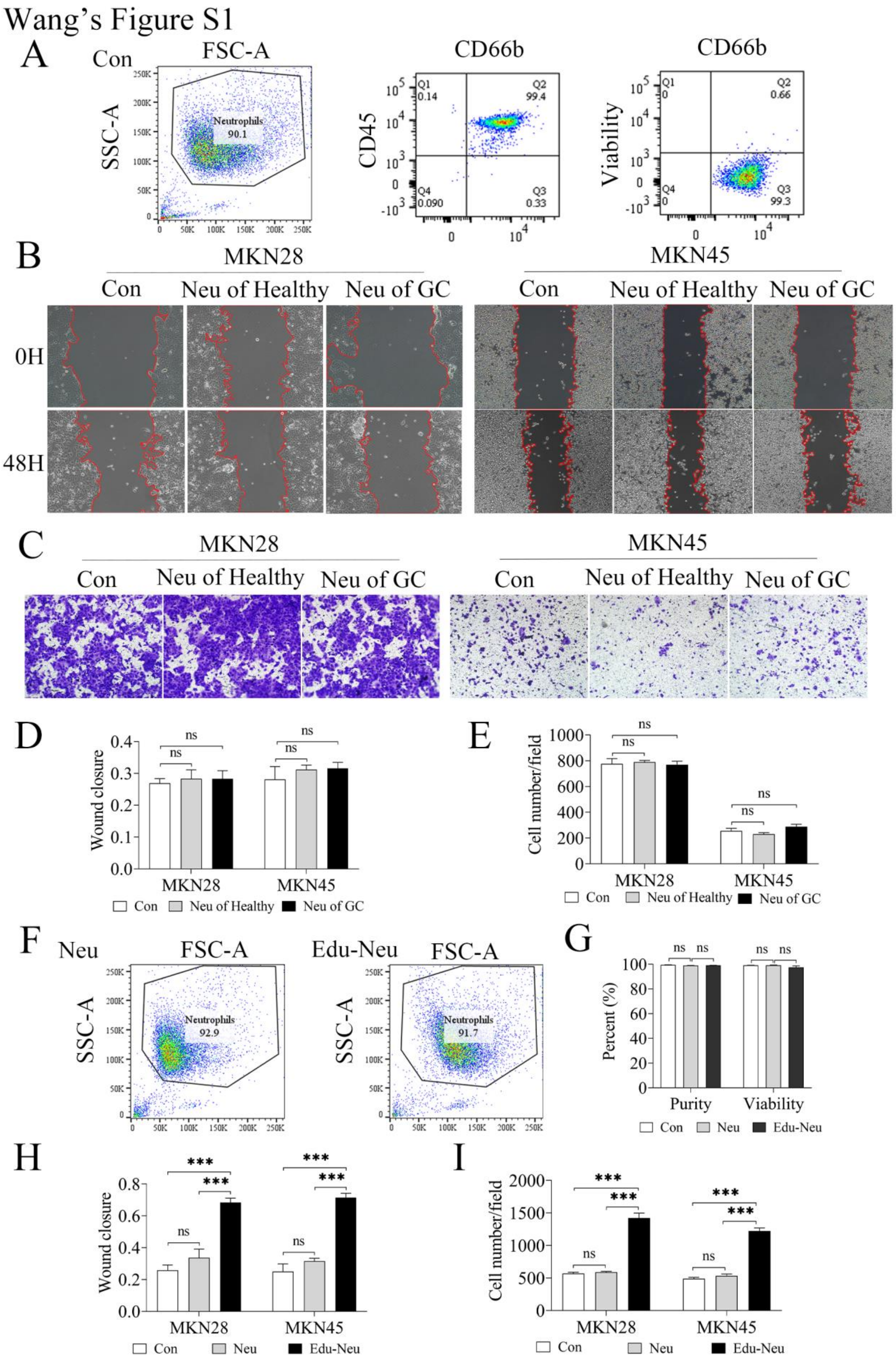

Figure s1. (**A**) FCM assays showed the isolated neutrophils had excellent purity and viability. (**B**,

C, **D**, **E**) CMs of isolated peripheral neutrophils from healthy donors (Neu of Healthy) and gastric cancer patients (Neu of GC) had no impact on MKN28 or MKN45 cells migration (**B**, **D**) and invasion (**C**, **E**) (×100). (**F**, **G**) FCM analyses showed co-culture with tumor cells exerted no remarkable influence on purity and viability of neutrophils. (**H**, **I**) CMs of Edu-Neus promoted MKN28 or MKN45 cell migration (**H**) and invasion (**I**). (***, P<0.001) (Neu, neutrophil; Edu-Neu, tumor-educated neutrophil)

Wang's Figure S2

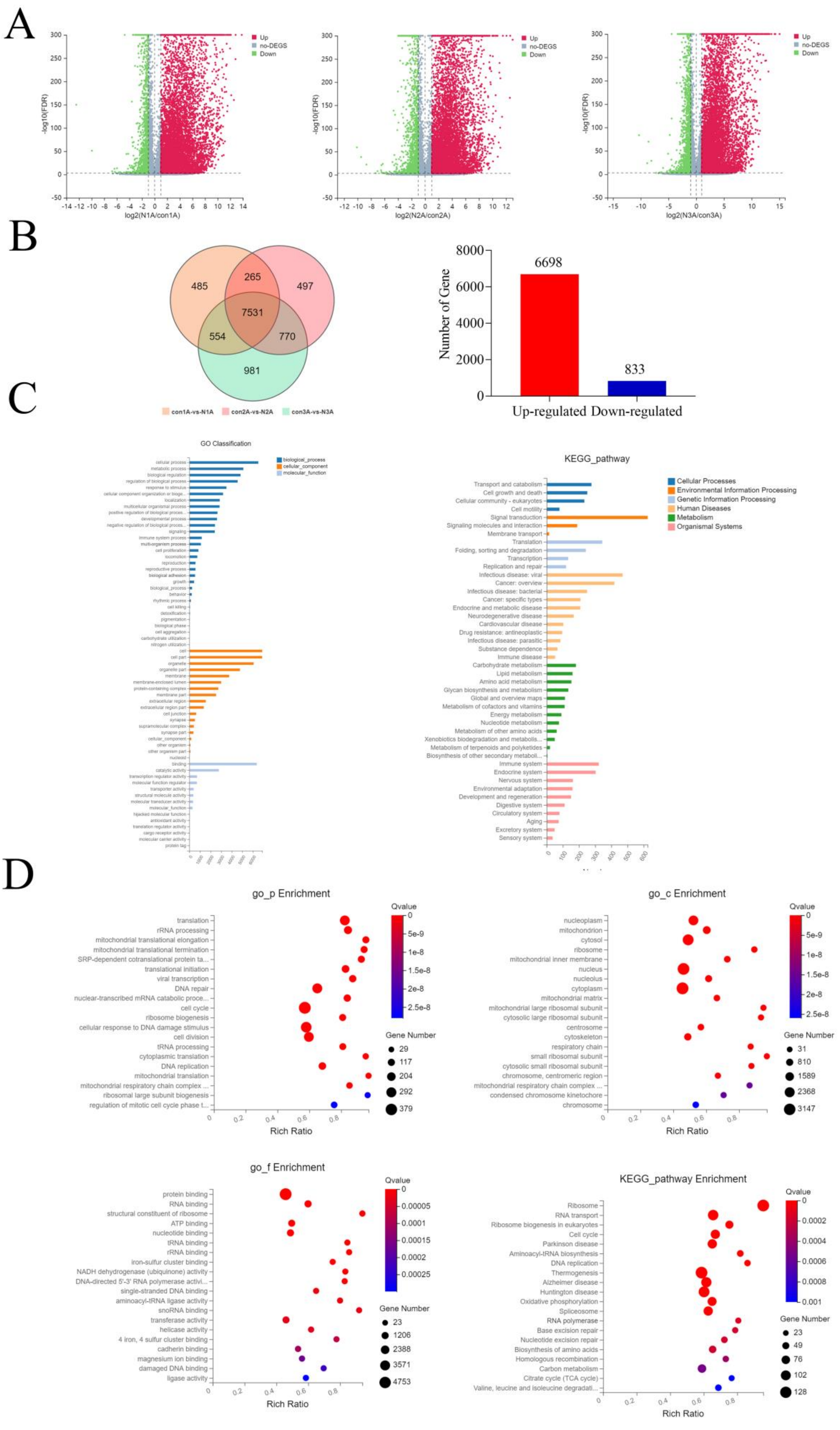

Figure s2. (**A**) Multiple genes were found to be differentially expressed in MKN45-educated neutrophils, including (7457, 7440, 7646) up-regulated genes and (1378, 1623, 2190) down-regulated genes in the three donors, respectively. (**B**) 7531 differential expressed genes (DEGs) overlapped in the three donors, including 6698 up-regulated genes and 833 down-regulated genes. (**C**, **D**) Gene Ontology (GO) analysis and KEGG pathway analysis.

Wang's Figure S3

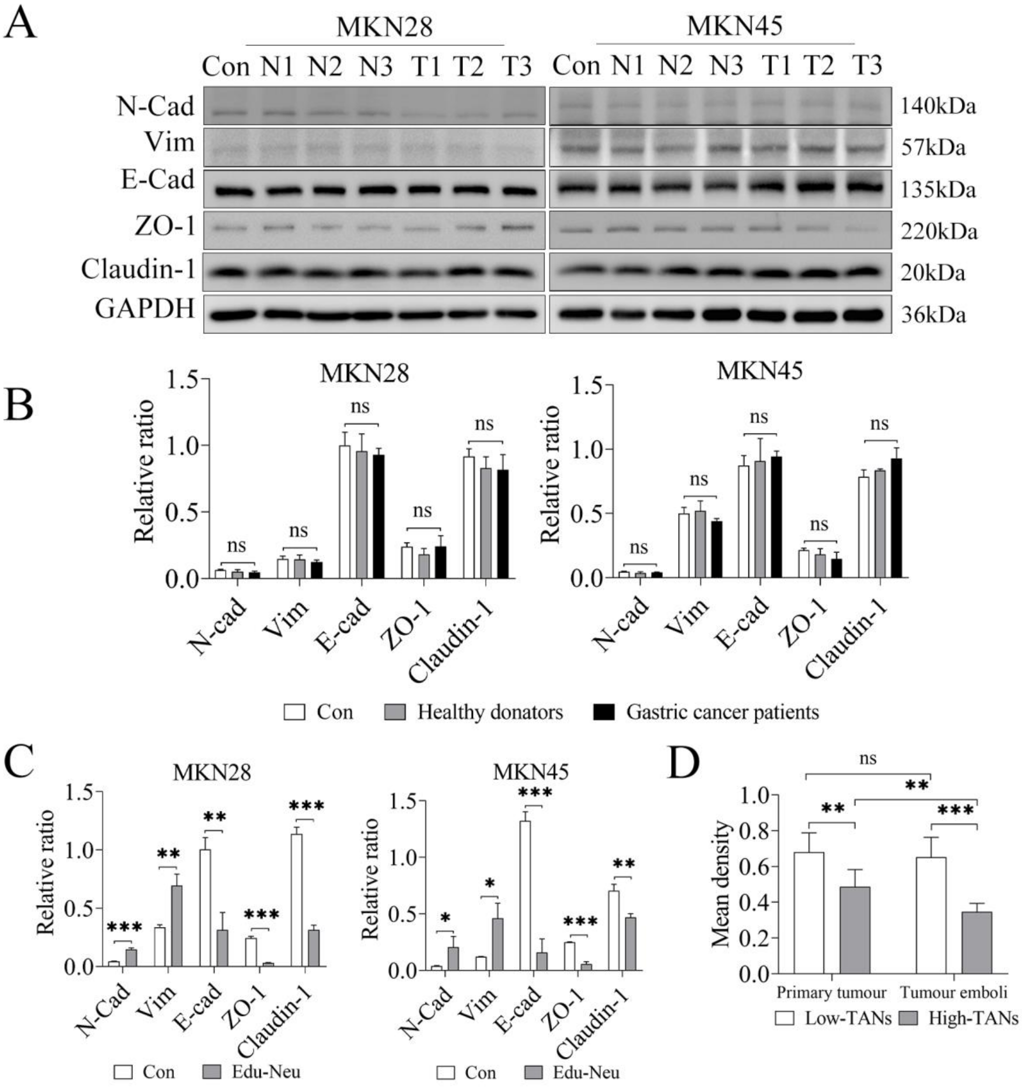

Figure s3. (**A**, **B**) CMs of isolated peripheral neutrophils from healthy donors (N1, N2 and N3) and gastric cancer patients (T1, T2 and T3) had no impact on the expression of proteins associated with EMT of MKN28 or MKN45 cells. (**C**) CMs of Edu-Neus decreased the expression of E-cad, ZO-1 and Claudin-1, while the expression of Vim and N-cad was increased. (**D**) E-cad expression in tumor tissues or lymphatic cancer emboli was decreased in high-TANs group compared with that in low-TNAs group, and E-cad levels in lymphatic cancer emboli were further decreased with regard to tumor tissues in high-TANs group. (*, P<0.05; **, P<0.01; ***, P<0.001) (Edu-Neu, tumor-educated neutrophil)

Wang's Figure S4

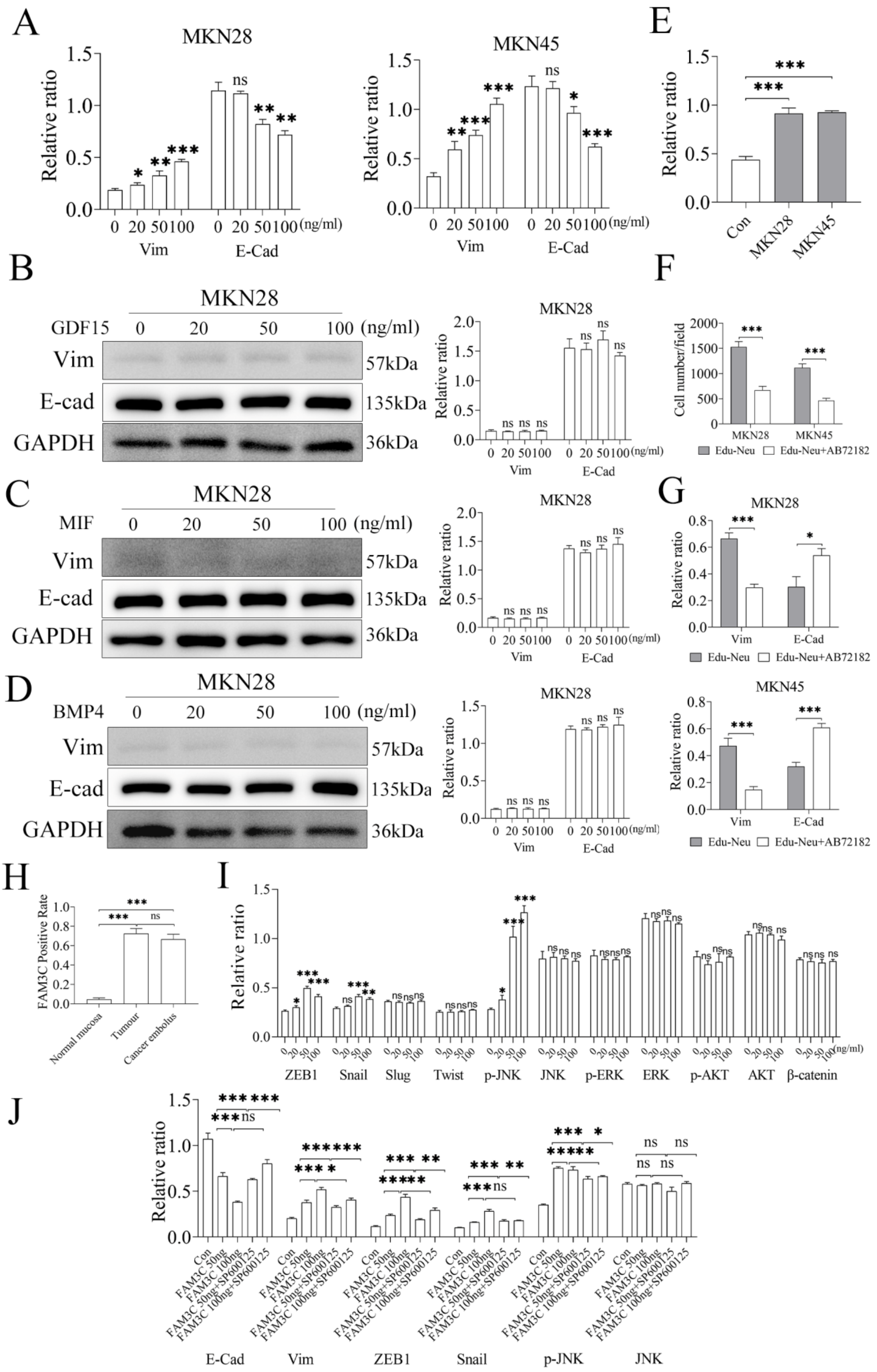

Figure s4. (**A**) FAM3C decreased E-cad expression and increased Vim expression of MKN45 and MKN28 cells in a dose-dependent manner. (**B**, **C**, **D**) GDF15 (**B**), MIF (**C**) and BMP4 (**D**) had no effects of E-cad and Vim expression. (**E**) Co-culture with MKN28 or MKN45 cells increased FAM3C levels in neutrophils. (**F**, **G**) Blockage of FAM3C with a neutralizing antibody reversed the enhanced-invasiveness (**F**) or induced-EMT (**G**) of tumor cells by Edu-Neus. (**H**) FAM3C positive rate were higher in TANs in human gastric tumor tissues and cancer emboli than that in normal stomach tissues by IHC assay. (**I**) FAM3C treatment up-regulated p-JNK as well as ZEB1 and Snail expression in MKN45 cells in a dose-dependent manner, but exerted no marked effects on expression of p-ERK, p-Akt, Slug and β-Catenin, and were reversed with JNK inhibitor treatment (**J**). (*, $P<0.05$; **, $P<0.01$; ***, $P<0.001$) (Edu-Neu, tumor-educated neutrophil)

Wang's Figure S5

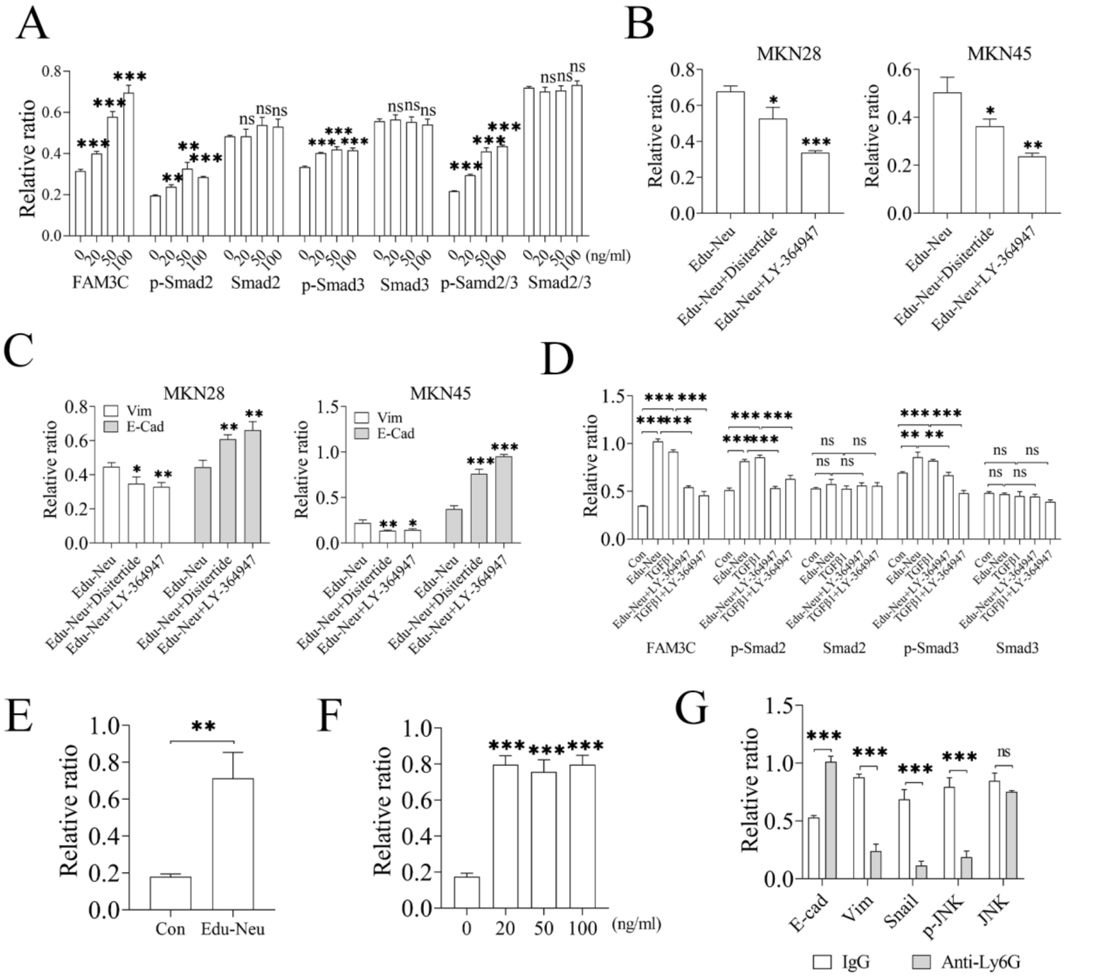

Figure s5. (**A**) TGFβ1 up-regulated FAM3C and p-Smad2, p-Smad3 or p-Smad2/3 expression in a dose-dependent manner. (**B**) Treatment with Disitertide or LY-364947 inhibited FAM3C expression. (**C**) Treatment with Disitertide or LY-364947 reversed the expression of EMT markers in tumor cells. (**D**) LY-364947 could down-regulate FAM3C and p-Smad2 or p-Smad3 expression in neutrophils. (**E**) Co-culture with neutrophils could increase CD151 expression. (**F**) FAM3C could increase CD151 expression in tumor cells. (**G**) E-cad level was increased whereas expressions of Vim, Snail and p-JNK were decreased significantly in anti-Ly6G-treated group with relative to IgG-treated group. (*, P<0.05; **, P<0.01; ***, P<0.001) (Edu-Neu, tumor-educated neutrophil)

Supplementary Table 1

Table s1A. Clinicopathologic features associated with LNM in T1b gastric cancer.

Clinicopathologic Features		LNM		χ^2	***P***
		**presence (n=61) (%)**	**absence (n=196) (%)**		
Gender	male	24 (32.4)	50 (67.6)	3.694	0.051
	female	37 (20.2)	146 (79.8)		
Age (year)	<65	26 (19.1)	110 (80.9)	2.882	0.078
	≥65	35 (28.9)	86 (71.1)		
Tumor location in the stomach	upper third	12 (18.5)	53 (81.5)	4.432	0.107
	middle third	12 (17.9)	55 (82.1)		
	lower third	37 (29.6)	88 (70.4)		
Tumor diameter (cm)	<2	18 (17.0)	88 (83.0)	4.803	0.090
	2-3	23 (27.4)	61 (72.6)		
	≥3	20 (29.9)	47 (70.1)		
Macroscopic type	elevated	8 (27.6)	21 (72.4)	1.011	0.620
	flat	3 (15.0)	17 (85.0)		
	depressed	50 (24.0)	158 (76.0)		
Depth of invasion	SM1	9 (13.0)	60 (87.0)	5.177	0.014
	SM2	52 (27.7)	136 (72.3)		
Lauren classification	intestinal	25 (15.3)	138 (84.7)	33.214	0.000
	diffuse	4 (14.3)	24 (85.7)		
	mixed	28 (57.1)	21 (42.9)		
	not defined	4 (23.5)	13 (76.5)		
Histological classification	well	2 (4.9)	39 (95.1)	18.700	0.000
	moderately	27 (20.6)	104 (79.4)		

	poorly	32 (37.6)	53 (62.4)		
Lymphatic invasion	absence	22 (11.5)	169 (88.5)	58.725	0.000
	presence	39 (59.1)	27 (40.9)		
Perineural invasion	absence	54 (22.4)	187 (77.6)	2.689	0.068
	presence	7 (43.8)	9 (56.3)		
H. pylori infection	absence	43 (23.1)	143 (76.9)	0.045	0.744
	presence	18 (25.4)	53 (74.6)		
TANs	low	17 (12.4)	120 (87.6)	19.476	0.000
	high	44 (36.7)	76 (63.3)		

Table s1B. Multivariate analysis of risk factors for LNM in T1b gastric cancer.

Clinicopathologic Features	Odds ratio	95% confidence interval	*P*
Depth of invasion	2.581	0.930-7.158	0.069
Mixed Lauren classification	8.676	1.906-39.492	0.007
Poorly differentiation	4.040	0.631-25.849	0.140
Lymphatic invasion	8.773	4.082-18.858	0.000
Higher TANs	3.519	1.606-7.710	0.002

Table s1C. Clinicopathologic features associated with LNM in SM1 gastric cancer.

Clinicopathologic Features		LNM		χ^2	*P*
		presence (n=9) (%)	absence (n=60) (%)		
Gender	male	4 (22.2)	14 (77.8)	0.880	0.226
	female	5 (9.8)	46 (90.2)		
Age (year)	<65	5 (12.8)	34 (87.2)	0.000	1.000
	≥65	4 (13.3)	26 (86.7)		

Tumor location in the stomach	upper third	0 (0.0)	16 (100.0)	4.686	0.102
	middle third	2 (9.1)	20 (90.9)		
	lower third	7 (22.6)	24 (77.4)		
Tumor diameter (cm)	<2	4 (12.9)	27 (87.1)	1.347	0.531
	2-2.9	4 (19.0)	17 (81.0)		
	≥3	1 (5.9)	16 (94.1)		
Macroscopic type	elevated	1 (10.0)	9 (90.0)	0.970	0.722
	flat	0 (0.0)	8 (100.0)		
	depressed	8 (15.7)	43 (84.3)		
Lauren classification	intestinal	2 (4.2)	46 (95.8)	16.675	0.000
	diffuse	0 (0.0)	5 (100.0)		
	mixed	7 (53.8)	6 (46.2)		
	not defined	0 (0.0)	3 (100.0)		
Histological classification	well	0 (0.0)	17 (100.0)	11.787	0.001
	moderately	2 (5.9)	32 (94.1)		
	poorly	7 (38.9)	11 (61.1)		
Lymphatic invasion	absence	4 (6.7)	56 (93.3)	12.463	0.001
	presence	5 (55.6)	4 (44.4)		
Perineural invasion	absence	9 (13.2)	59 (86.8)	0.000	1.000
	presence	0 (0.0)	1 (100.0)		
H. pylori infection	absence	8 (17.0)	39 (83.0)	1.104	0.254
	presence	1 (4.5)	21 (95.5)		
TANs	low	1 (2.6)	37 (97.4)	6.170	0.009
	high	8 (25.8)	23 (74.2)		

Table s1D. Multivariate analysis of potential risk factors for LNM in the patients with SM1 gastric cancer.

Clinicopathologic Features	Odds ratio	95% confidence interval	*P*

Lymphatic invasion	11.895	0.791-178.891	0.073
TANs	5.763	0.382-86.933	0.206

Table s1E. Clinicopathologic features associated with LNM in SM2 gastric cancer.

Clinicopathologic Features		LNM		χ^2	***P***
		**presence (n=52) (%)**	**absence (n=136) (%)**		
Gender	male	20 (35.7)	36 (64.3)	2.045	0.113
	female	32 (24.2)	100 (75.8)		
Age (year)	<65	21 (21.6)	76 (78.4)	3.024	0.073
	≥65	31 (34.1)	60 (65.9)		
Tumor location in the stomach	upper third	12 (24.5)	37 (75.5)	1.667	0.468
	middle third	10 (22.2)	35 (77.8)		
	lower third	30 (31.9)	64 (68.1)		
Tumor diameter (cm)	<2	14 (18.7)	61 (81.3)	5.952	0.049
	2-3	19 (30.2)	44 (69.8)		
	≥3	19 (38.0)	31 (62.0)		
Macroscopic type	elevated	7 (36.8)	12 (63.2)	1.019	0.636
	flat	3 (25.0)	9 (75.0)		
	depressed	42 (26.8)	115 (73.2)		
Lauren classification	intestinal	23 (20.0)	92 (80.0)	19.498	0.000
	diffuse	4 (17.4)	19 (82.6)		
	mixed	21 (58.3)	15 (41.7)		
	not defined	4 (28.6)	10 (71.4)		
Histological classification	well	2 (8.3)	22 (91.7)	7.975	0.017
	moderately	25 (25.8)	72 (74.2)		
	poorly	25 (37.3)	42 (62.7)		

Lymphatic invasion	absence	18 (13.7)	113 (86.3)	39.573	0.000
	presence	34 (59.6)	23 (40.4)		
Perineural invasion	absence	45 (26.0)	128 (74.0)	2.001	0.128
	presence	7 (46.7)	8 (53.3)		
H. pylori infection	absence	35 (25.2)	104 (74.8)	1.198	0.265
	presence	17 (34.7)	32 (65.3)		
TANs	low	16 (16.2)	83 (83.8)	12.630	0.000
	high	36 (40.4)	53(59.6)		

Table s1F. Multivariate analysis of potential risk factors for LNM in the patients with SM2 gastric cancer.

Clinicopathologic Features	Odds ratio	95% confidence interval	*P*
Tumor size≥3cm	2.270	0.805-6.404	0.121
Mixed Lauren classification	6.506	1.259-33.614	0.025
Poorly differentiation	3.134	0.465-21.108	0.240
Lymphatic invasion	7.421	3.260-16.894	0.000
TANs	3.518	1.522-8.129	0.003

Supplementary Table 2

Table s2A. Multivariate analysis of potential risk factors for lymphatic invasion in patients with T1b gastric cancer.

**Clinicopathologic Features**	**Odds ratio**	**95% confidence interval**	***P***
Age	1.956	0.991-3.860	0.053
Tumor location in the stomach	3.119	1.290-7.543	0.012
Tumor size	3.849	1.672-8.860	0.002
Depth of invasion	2.979	1.255-7.069	0.013
Lauren classification	3.901	0.823-18.481	0.086
Histological classification	7.464	1.386-40.210	0.019
TANs	2.467	1.279-4.756	0.007

Table s2B. Multivariate analysis of potential risk factors for lymphatic invasion in the patients with SM1 gastric cancer.

**Clinicopathologic Features**	**Odds ratio**	**95% confidence interval**	***P***
Tumor diameter	8.512	0.587-123.510	0.117
Histological classification	2.352	0.364-15.221	0.369
TANs	15.856	1.536-163.661	0.020

Table s2C. Multivariate analysis of potential risk factors for lymphatic invasion in the patients with SM2 gastric cancer.

**Clinicopathologic Features**	**Odds ratio**	**95% confidence interval**	***P***
Tumor size	3.515	1.533-8.056	0.003
Histological classification	5.054	1.044-24.462	0.044
TANs	2.243	1.148-4.382	0.018

Table s2D. Neutrophils in lymphatic cancer emboli were associated with LNM in the T1b or SM2 gastric cancer.

**Clinicopathologic Features**		**LNM of T1b tumors**				**LNM of SM2 tumors**			
		**presence (n=39) (%)**	**absence (n=27) (%)**	**χ^2**	***P***	**presence (n=34) (%)**	**absence (n=23) (%)**	**χ^2**	***P***
Neutrophils in lymphatic cancer embolus	Absence	15 (42.86)	20 (57.14)	6.757	0.006	13 (41.94)	18 (58.06)	7.320	0.003
	Presence	24 (77.42)	7 (22.58)			21 (80.77)	5 (19.23)		
	—Less	16 (84.21)	3 (15.79)	0.486	0.384	15 (88.24)	2 (11.76)	0.647	0.302
	—More	8 (66.67)	4 (33.33)			6 (66.67)	3 (33.33)		

Table s2E. Correlation of TANs abundance in tumors with neutrophils in lymphatic cancer emboli in T1b or SM2 gastric cancer.

		TANs		*χ2*	*r*	*P*
		Low	High			
lymphatic invasion of T1b	Absence	114 (59.7)	77 (40.3)	11.179	0.217	0.001
	Presence	23 (34.8)	43 (65.2)			
lymphatic invasion of SM2	Absence	77 (58.8)	54 (41.2)	5.705	0.186	0.012
	Presence	22 (38.6)	35 (61.4)			
neutrophils in lymphatic cancer embolus of T1b tumors	Absence	19 (54.3)	16 (45.7)	10.644	0.433	0.001
	Presence	4 (12.9)	27 (87.1)			
neutrophils in lymphatic cancer embolus of SM2 tumors	Absence	18 (58.1)	13 (41.9)	9.142	0.437	0.001
	Presence	4 (15.4)	22 (84.6)			

Table s3. Gene alterations associated with N1 or N2 phenotype in Edu-Neus

N1 (tumor-suppressive)			N2 (tumor-promoting)		
**Gene**	**log2 (Edu-Neus/Con)**	***P***	**Gene**	**log2 (Edu-Neus/Con)**	***P***
FAS	-1.554	0.000	Adenosine	undetected	
Granzyme			Arginase 1	undetected	
B	undetected				
INF-β	undetected		BV8	undetected	
INF-γ	undetected		CCL17	undetected	
LPS	undetected		CCL2	-3.764	0.000
MET	9.436	0.000	Elastase	undetected	
			G-CSF	undetected	
			Hydrogen	undetected	
			peroxide		
			ICAM1	undetected	
			IL10	-1.503	0.000
			IL17D'	3.391	0.000
			IL-1β	undetected	
			IL8	undetected	
			L-lactate	undetected	
			LOX-1	undetected	
			MMP9	undetected	
			MPO	undetected	
			mPR3	undetected	
			NAMPT	undetected	
			NETs/PAD4	undetected	
			Oncostatin M	2.476	0.000
			PDGFB	4.370	0.000
			PGE2	undetected	
			ROS	undetected	

	S100A8/9	-1.088	0.000
	STAT/IRF8	undetected	
	STAT3	undetected	
	TGFB3	4.099	0.000
	TGFBRAP1	2.335	0.000
	TNF-a	undetected	
	TRAIL	undetected	
	VEGFB	2.151	0.000
